# *In Silico* Prediction and Analysis of *Caenorhabditis* EF-hand Containing Proteins

**DOI:** 10.1371/journal.pone.0036770

**Published:** 2012-05-25

**Authors:** Manish Kumar, Shadab Ahmad, Ejaz Ahmad, Muheet Alam Saifi, Rizwan Hasan Khan

**Affiliations:** 1 Advanced Instrumentation Research Facility, Jawaharlal Nehru University, New Delhi, India; 2 Centre for Computational Biology and Bioinformatics, Jawaharlal Nehru University, New Delhi, India; 3 Interdisciplinary Biotechnology Unit, Aligarh Muslim University, Aligarh, India; 4 Department of Zoology, College of Science, King Saud University, Riyadh, Kingdom of Saudi Arabia; Wayne State University School of Medicine, United States of America

## Abstract

Calcium (Ca^+2^) is a ubiquitous messenger in eukaryotes including *Caenorhabditis*. Ca^+2^-mediated signalling processes are usually carried out through well characterized proteins like calmodulin (CaM) and other Ca^+2^ binding proteins (CaBP). These proteins interact with different targets and activate it by bringing conformational changes. Majority of the EF-hand proteins in *Caenorhabditis* contain Ca^+2^ binding motifs. Here, we have performed homology modelling of CaM-like proteins using the crystal structure of *Drosophila melanogaster* CaM as a template. Molecular docking was applied to explore the binding mechanism of CaM-like proteins and IQ1 motif which is a ∼25 residues and conform to the consensus sequence (I, L, V)QXXXRXXXX(R,K) to serve as a binding site for different EF hand proteins. We made an attempt to identify all the EF-hand (a helix-loop-helix structure characterized by a 12 residues loop sequence involved in metal coordination) containing proteins and their Ca^+2^ binding affinity in *Caenorhabditis* by analysing the complete genome sequence. Docking studies revealed that F165, F169, L29, E33, F44, L57, M61, M96, M97, M108, G65, V115, F93, N104, E144 of CaM-like protein is involved in the interaction with IQ1 motif. A maximum of 170 EF-hand proteins and 39 non-EF-hand proteins with Ca^+2^/metal binding motif were identified. Diverse proteins including enzyme, transcription, translation and large number of unknown proteins have one or more putative EF-hands. Phylogenetic analysis revealed seven major classes/groups that contain some families of proteins. Various domains that we identified in the EF-hand proteins (uncharacterized) would help in elucidating their functions. It is the first report of its kind where calcium binding loop sequences of EF-hand proteins were analyzed to decipher their calcium affinities. Variation in Ca^+2^-binding affinity of EF-hand CaBP could be further used to study the behaviour of these proteins. Our analyses postulated that Ca^+2^ is likely to be key player in *Caenorhabditis* cell signalling.

## Introduction

All living cells are required to have a cross talk with the environment where they reside. This cellular communication is facilitated by a lot of messengers. Calcium (Ca^+2^) is one of the most important second messengers that is involved in numerous signal transduction processes in eukaryotes [1]. Intracellular Ca^+2^ is implicated in a variety of cellular functions in eukaryotes including cell migration, contraction, secretion, proliferation and differentiation, exocytosis, transcellular ion transport, neurotransmitter release and gap junction regulation [2], [3]. The Ca^+2^ concentration in the cytoplasm is kept in the nanomolar range whereas in the sub cellular compartments it is in the milli molar range [1]. In response to external stimuli (hormone, stress etc.), there is transient increase in the cytoplasmic Ca^+2^ concentration. Transient elevation of Ca^+2^ is perceived by two classes of proteins: Ca^+2^ sensors and Ca^+2^ buffers [2]. Association of Ca^+2^ with the Ca^+2^ sensors (e.g. calmodulin, troponin C) leads to the conformational change, resulting in alteration of its activity or its ability to interact with other proteins or nucleic acids. Interaction of Ca^+2^ sensors with its target results in the modulation of target's function or activity [2], [4]. Ca^2+^ buffers (e.g., calbindin D9K and parvalbumin) are a smaller subset of the EF-hand protein family which does not undergo a significant conformational change on binding to free Ca^2+^ [5], [6]. These proteins respond to the Ca^2+^ signal either by transmitting the signal throughout the cell or helping the cell in getting rid of the free Ca^2+^ from the cytoplasm [5], [6]. One of the well-studied CaBP is calmodulin (CaM), a four EF-hand highly conserved CaBP [4].

In the EF-hand family of proteins the functional unit is a pair of EF-hand motifs [7]. In an EF-hand motif there are three parts namely helix, loop and helix. The helix-loop-helix is usually 29 residues long. The first helix which is at N terminal and known as E consist of 10–12 residues (1–10). Next to helix E, is the Ca^+2^ coordination loop that has 12 residues (10–21). The last part is helix F that is at C terminal and consists of 10–12 residues (19–29). Structurally the two helices (alpha) E and F are 90 degree to each other. Residue 1 is often Glu (E) and a Gly (G) at residue 15 is highly conserved, as is Ile (I) at residue 17. The name EF-hand is derived from the three-dimensional arrangement of helix, loop and helix, which gives an impression of the thumb, index and middle fingers of a hand [7].

The canonical EF-hand motif, which has been most extensively studied, is highly conserved in the Ca^+2^ coordination loop at position 1, 3, 5, 7, 9, and 12. These residues offer Oxygen for Ca^+2^ binding. At position 12 of Ca^+2^ coordination loop generally glutamate is found that offers both its side-chain oxygen for Ca^+2^ coordination. Out of seven ligands used for the coordination with the Ca^+2^, six are given by twelve-residue loop (loop sequence positions 1, 3, 5, 7, 9 and 12) and seventh is the water molecule. Residues 1, 3, 5 and 12 offer side chain carboxy group for coordination with the Ca^+2^ and residue at 7 coordinates through backbone carbonyl group. Residue 9 forms link with Ca^+2^ through water bridge [8]. There is a lot of variation in the Ca^+2^ binding constant in different CaBP and might be having some correlation with the sequence position at 1, 3, 5, 7, and 9.

In most of the cases functional unit of EF-hand proteins is a pair of EF-hand motifs and proteins with four EF-hands usually have one pair of EF-hands in one domain and the other pair in second domain. Calpain is an exception to EF-hand pairing rule. It comprises of two subunits with each subunit having five EF-hands. The unpaired EF-hand in subunits pair to form heterodimer [9]. Large EF-hand super family of proteins has been divided into 66 sub families [10]. The classification of EF-hand proteins into subfamilies has been done the basis of differences in the number of EF-hand pairs, organization of EF-hand pairs sequences of amino acids within or outside the EF-hand motif, affinity for Ca^+2^ and affinity for target proteins [10].

CaM-like protein from *C. elegans*, may bind to IQ motifs of myosin. Myosins, neuronal growth proteins, voltage-gated channels and certain signalling molecules contain IQ motifs [11] that can bind to either CaM or CaM-related proteins. IQ motifs are of ∼25 amino acids in length and conform to the consensus sequence (I, L,V)QXXXRXXXX(R,K) [11]. In order to understand binding of CaM to IQ motifs and the nature of the interactions, we have docked the IQ1 motif of myosin V from *Saccharomyces cerevisiae* (Myo2P) on CaM-like protein and studied three-dimensional structure of the complex between IQ1 and CaM (IQ1-CaM-like protein).

Several EF-hand proteins have been identified in *Caenorhabditis* genus including Calcineurin, CaM [12] and Ced4 [13]. Calcineurin B is Ca^+2^/CaM-dependent serine/threonine protein phosphatase [14]. All these proteins have been identified through different experimental techniques. Many CaBP that do not have EF-hand also bind to Ca^+2^ [15]. Numerous CaM binding proteins have also been isolated from *C. elegans*. Phosphodiesterase, phosphatase, kinases and myosins have been shown to bind CaM [16]. Identifying and characterizing EF-hand proteins encoded in the *Caenorhabditis* genome would give a deeper understanding of the biology of proteins. Functional and structural classification of EF-hand containing proteins can be a first step in the identification of CaBP that might be involved in cellular process. Keeping this in view we thoroughly analysed the *Caenorhabditis* genome for genes encoding proteins with EF-hand motifs.

## Results

### Structural analysis

**Figure 1:**
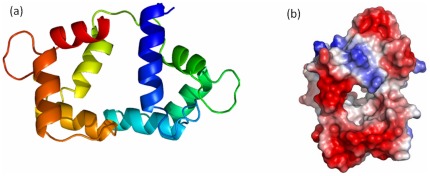
Homology modelled CaM-like protein. (a) Cartoon representation of model of CaM-like protein. The N& C terminal is dark blue and dark red respectively (b) Electrostatic molecular surface representation of the model of CaM-like protein.

**Figure 2:**
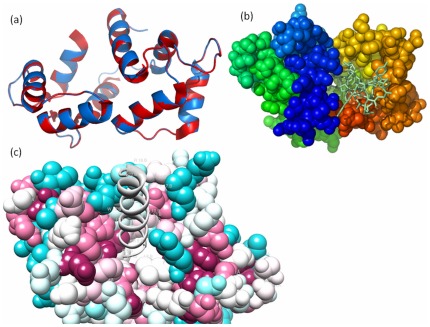
CaM-like modelled protein with its ligand IQ1 motif. (a) Superimposed structures of theoretical 3D model of the CaM-like protein (with C-alpha atoms only) on its respective template in cartoon representation. Template is in blue colour and model is in red colour; (b) CaM-like protein in complex with IQ1 motif. The ligand IQ1 motif is shown as grey stick; (c) Dark violet colour is for the most conserved. The ligand IQ1 motif is shown as grey ribbon.

Comparative modeling of CaM-like protein (gi: 37699821) of *C. elegans* was carried out using Modeller program. For modeling of CaM-like protein, crystal structure of *Drosophila melanogaster* CaM was used as a template having PDB ID 2BKH|B [17]. The sequence alignment of target and template for CaM-like protein (gi: 37699821) was about 65% (Table 1). Individual alignment was given as input to Modeller to build 3D structures, and the resulting 3D structure was evaluated using Verify3D and PROCHECK. Structure was deposited in the protein model database and the structure was accepted with less than 3% stereochemical check failures (Figure 1a). The PMDB ID of CaM-like protein 3D model is PM0077671. This analysis has led to the conclusion that the models are quiet reliable. Homology model of CaM-like protein consists of two domains that is N and C terminal domain (Figure 1a). Each domain has a pair of EF-hands and the loop of EF-hand might coordinate with Ca^+2^ ions (Figure 1a). CaM-like protein model is having very less deviation with respect to the template (Figure 2a and Table 2). Overall structural fold in CaM-like protein model is almost identical to the template. As Drosophila CaM binds well with Ca^+2^, it is important to analyze and characterize the predicted structure for affinity to Ca^+2^. Ca^+2^ binding motif of model was found to be having good Ca^+2^ binding affinity [18]. IQ1 motif of myosin V from *Saccharomyces cerevisiae* having AILLQTNIRALWKREYYRAA [19] sequence was used for docking. IQ1 motif of myosin V from *Saccharomyces cerevisiae* was modelled with PEP-FOLD [20]. PEP-FOLD is a *de novo* approach for predicting peptide structures from amino acid sequences. Structural studies have revealed that IQ motif containing proteins like myosin interact with CaM [21]. Modelled IQ1 motif of myosin V from *Saccharomyces cerevisiae* (Myo2P) was docked to the model CaM-like protein, keeping CaM-like protein as a fixed molecule, using GRAMM-X (Figure 2b). Program GRAMM (Global Range Molecular Matching) performs an exhaustive 6-dimensional search through relative translations and rotations of the molecules. Best docked structure in terms of score was analysed in detail. From the docked model, it is inferred that IQ1 motif form three hydrogen bonds with CaM-like protein model (Figure 2b). H-bonds occur between A1 (IQ1 motif) and A112 (model), T6 (IQ1 motif) and A39 (model), N7 (IQ1 motif) and E36 (model) with 3.21A°, 3.31A° and 3.09A° distance respectively. Closer molecular interaction between CaM-like protein and IQ1 motif is seen among CaM-like protein residues F165, F169, L29, E33, F44, L57, M61, M96, M97, M108, G65, V115, F93, N104, E144, and IQ1 motif residues A1, I2, L3, L4, Q5. Interaction between CaM and IQ1 motif is a mixture of polar and non polar interactions. Non polar interactions have a major role here. In order to know conservation of the modelled residues, model pdb file was subjected to ConSurf [22] analysis. The Table 3 gives modelled residues position (not sequence position) and conservation score. It has been shown in the figure that IQ1 motif is docked in a place where good number of residues are conserved (dark violet colour represent conservation) (Figure 2c). The model contains favourable binding sites like groove lined by polar and non polar residues. It is known that CaM interacts with myosin and regulates its activity [23]. In the same way it could be said that *C. elegans* CaM-like protein might be regulating the myosin network in *C. elegans*.

**Table 1:** Template and Target the sequences used for modelling.

Name of the model (Target) & organism	Model Accession no. & Amino acid range used	Template PDB ID & Amino acid range used	Percentage (%) Sequence identity with template
CaM-like protein *(C. elegans)*	gi37699821	2BKH|B	65
	(28–171)	(4–147)	

**Table 2:** Comparison of models with the template.

Name of the Protein	Template PDB ID	Number of C_α_ residues	RMS deviation of C_α_ of the model with the template
CaM-like protein	2BKHB	126	0.056 A°

**Table 3:** Conservation score of CaM like Protein model (9-conserved, 1- variable).

Modelled Residue Position	Sequence	Conservation Score	Residue Variety
17	F	8	F,I,Y,L
18	D	9	T,D,R,E
29	E	9	Q,D,K,E
30	L	9	F,L
34	M	8	M,I,L,V
35	R	8	Q,T,K,R,G
36	S	8	H,A,S,M,T,N,G
37	L	9	F,M,I,L
38	G	8	D,G,E,V
41	P	9	A,I,P,L
61	I	8	T,I,L,V
62	D	5	S,N,D,G,E
63	F	8	S,F,W,E,L,Y
65	E	9	A,S,D,K,E
66	F	9	S,F,L,Y
85	A	9	S,A,T,G,V
86	F	9	F
89	F	9	F,L
90	D	9	A,D,E
125	I	8	A,I,V
126	D	9	S,N,D
128	D	8	M,N,D
130	D	8	S,N,D,G,E
131	G	8	S,N,K,G,E

### Identification of EF-hands

In order to have a clear understanding about the nature of Ca^+2^ signalling pathways and Ca^+2^ sensors present in *Caenorhabditis*, identification of EF-hand using the *Caenorhabditis* genome sequences was undertaken. To identify EF-hand containing protein *Caenorhabditis* genome, protein sequences were retrieved from NCBI Protein database. Each protein sequence was then subjected to InterProScan [24] for identification of an EF-hand and other motifs. There are many databases for analyzing the protein sequences. InterProScan was chosen because it uses more than seven databases to analyze a sequence. Therefore assignment of a particular domain to a protein sequence by InterProscan is equivalent to assignment by more than seven databases. Protein sequences, which were shown not to have EF-hand domain, were not included in the list of EF-hand proteins. We have surveyed *Caenorhabditis* genome, for EF hand containing proteins using sensitive sequence profile matching algorithms (PSI-BLAST, BlastP). The protein sequence of *Caenorhabditis* CaM was used as a template in these searches. Search procedures such as PSI-BLAST have been used at E-value cut off of 0.0001 [25]. The output was checked for the presence of EF-hand domain using InterProScan. Sequence similarity search using well annotated EF-hand and CaBP of *Caenorhabditis* was also done. A large number of EF-hand proteins were identified by these methods. We were able to decipher a substantial number of EF-hand proteins which were not even mentioned in the Uniprot. These protein sequences were given their respective NCBI gi number. While curating our data utmost care was taken to include mostly those proteins which were identified as EF-hand proteins by more than one database. As a result of this criteria used in the identification of EF-hand, there is remote possibility of selecting a false positive. Published literatures were also thoroughly searched for the report of EF-hand proteins in *Caenorhabditis*. All these search together lead to making of a comprehensive repository of EF-hand proteins which includes not only well known EF-hand proteins but also those that were not mentioned in other databases. In this analysis an attempt was made to not only to identify EF-hand proteins but also to identify non EF-hand Ca^+2^/metal binding proteins. Rationale for this approach was to identify all those proteins that may play an important role as Ca^+2^/metal sensors and transducing molecules due to the presence of a number of Ca^+2^/metal binding domains. We have been able to identify 170 EF-hand proteins and 39 non EF-hand proteins with Ca^+2^/metal binding ability (Table S1). Overall we analysed 209 sequences either for the presence of Ca^+2^/metal binding domain or EF-hand domain. Tables S2 and Table 4 describe, function of EF-hand and non EF-hand protein with a detailed description of their functional characterization (whether function has been derived through electronic annotation or experiments like mutant phenotype etc.). The function of protein was also inferred by scanning UniProt and WORM database.

**Table 4:** Occurrence of EF-hand proteins in more than one *Caenorhabditis* species.

Predicted name	Length	Organism
CRE-CAL-2	171	*C. briggsae* (A8WSI9), *C. remanei* (E3LG34)
CaM like protein (CAL-1)	161	*C. briggsae* (A8Y3Q7), *C. elegans* (P04630)
CBN-CAL-4	182	*C. briggsae* (A8X6C6), *C. brenneri* (G0NRZ9), *C. Remanei* (E3M5L0)
CBR-TNC-2	160	*C. remanei* (E3LFS7), *C. brenneri* (G0NT97), *C. briggsae* (A8WSS9)
Hypothetical protein CBG18252	142	*C. briggsae* (A8XS91), *C. remanei* (E3LYJ5)
CNB-1	171	*C. brenneri* (G0P6N0), *C. briggsae* (A8Y4I1), *C. elegans* (Q20804)
MLC-3	153	*C. elegans* (B6EU49), alkali myosin light chain long isoform, *C. elegans* (P53014)
CBR-NCS-2	190	*C. elegans* (P36609), *C. brenneri* (G0MFI7), *C. remanei* (E3M356), *C. briggsae* (A8WWQ4)
CBG09836	196	*C. remanei* (E3LRU2), *C. briggsae* (A8X9R8)
CBR-PAT-10	161	*C. elegans* (P91328), *C. brenneri* (B3GDA0), C. Briggsae (A8XBR6)
CBR-CMD-1	149	*C. elegans* (O16305), *C. brenneri* (G0PHL7), *C. briggsae* (A8WPJ8)
CBG11969	81	*C. elegans* (P91423), *C. remanei* (E3LLY7), *C. brenneri* (G0PHP6), *C. briggsae* (A8XEQ0)

### Ca^+2^/metal binding proteins with no recognized EF-hand

Table S2 lists the proteins which contain Ca^+2^/metal binding domain but do not contain EF-hand domain. These sequences were retrieved from sequence similarity search. When these sequences were subjected to InterProScan, either it didn't identify any EF-hand domain or more than one database didn't confirm the presence of EF-hand domain. These proteins were neither included in the total number of EF-hand proteins nor in phylogenetic analysis.

This group has good number of proteins that possess zinc finger domain like the CBR-DYB-1 (A8Y1Z6) [26] of *C. Briggsae*. PROSITE documentation for ZZ zinc finger describes that it contains 7 positions coordinating with one zinc atom and out of the 7 positions 4 are completely conserved. This motif is also present in the adaptor proteins CBP and P300 in a region which is known to interact with YY1, E1A and TFIIB [27].

There is also one class of proteins that has phosphatidylinositol-specific phospholipase C (PLC) profiles. CBR-PLC-1 (A8XRA5) [26] of *C. Briggsae* has PLC domain. Phosphatidylinositol-specific PLC, a eukaryotic intracellular enzyme, plays an important role in signal transduction [28]. It catalyzes the hydrolysis of 1-phosphatidyl-D-myo-inositol-4,5-diphosphate into diacylglycerol and inositol-1,4,5-triphosphate. Reversible phosphorylation and association of regulatory proteins control this catalytic process. Through electronic annotation most of the proteins present in this table has been shown to bind Ca^+2^. The worm base and uniprot databases were also used to infer the functions for these proteins. Most of these functions have been proven through experiments like mutant phenotype etc. Functions range from development to neurological. Egg laying defective protein-8 (Q95X29) of *C. elegans* is involved in the embryo development, signalling pathways and regulation of mating behaviour [29]. All these functions have been proven through experimental means.

### Number of EF-hands

**Figure 3:**
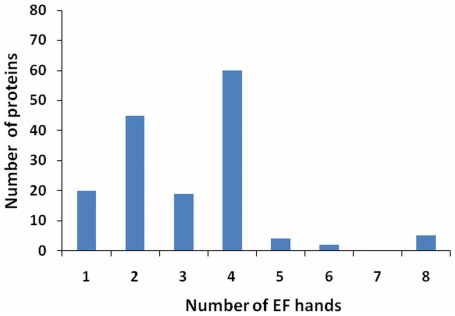
Occurrence of EF-hands. The number of *Caenorhabditis* proteins having 1, 2, 3, 4, 5, 6 or 8 EF-hands.

The number of EF-hands in each protein varied from one to eight. Most of these proteins have a pair of EF-hands which establishes their functional relevance [10]. There are many proteins with an odd number EF-hands i.e 1, 3, 5 (Figure 3). With regard to functionality of the odd number of EF-hands is concerned there exists, large number of possibilities. In order to fulfill the EF hand pairing criteria in EF hand protein with odd number of EF hands, they might function as homodimer or heterodimer. They may pair with other molecule (identical molecule) and form homodimer as in case of S100B [30]. There is also a possibility that two different domains of two identical molecules pair and form heterodimer like calpain [9].

Genomes of all the species of *Caenorhabditis* were scanned for the presence of Ca^+2^ dependent protein kinase having EF-hand but could not find out any. There is an evidence of Ca^+2^/CaM-dependent protein kinase II in one of the species of *Caenorhabditis* that is *C. elegans* but it does not have EF-hand [31]. These kinases are well regulated by CaM which is a well characterized four EF-hands protein. More than five troponin C are present in *Caenorhabditis* genome. Troponin C is part of troponin I and troponin T complex which regulates muscle contraction. In one of the species of *Caenorhabditis* that is *C. elegans*, troponin C isoform 2 (Q09665) has four EF-hands but it has been found out that there are only two Ca^+2^ binding EF-hands [32], [33]. CaM, proteins closely related to CaM and good number of hypothetical proteins has four EF-hands (Table S1). There is more than two neuronal calcium sensors-2 (NCS-2) [34] in *Caenorhabditis* genome which has three EF-hands. Myosin light chain regulatory protein (MLC-3), Spectrin protein [35] and NCS-3 [34] have two EF-hands (Table S1). MLC-4 [36] and receptor mediated endocytosis-1 (RME-1) [37] have single EF-hand (Table S1). Most of the proteins belonging to the category of 5, 6, 8 EF-hands are not characterized by experiments.

### Identification of other domains in EF-hand containing proteins

Table S1 lists other domains found in the EF-hand containing protein. In these EF hand proteins position of the EF hand is not confined to one part of the protein, it could be at one end or the other, or in the middle of the protein with enzymatic or regulatory domains either following or preceding the EF-hand domain.

Some of the EF hand proteins have Eps15 homology domain [37]. Eps15 repeats are protein-protein interaction module of about 95 residues long. It was first seen in tyrosine kinase substrate Eps15 and 15R [38]. The unique aspect of Eps15 domain is that a part of Eps15 domain may act as a Ca^+2^ binding domain EF-hand type.

Several identified domains like PLC [39] and pleckstrin homology [40] indicate involvement of EF hand protein in the membrane transport and calcium signalling network. PLC is a family of enzymes catalyzing the hydrolysis of phosphatidylinositol 4, 5-bisphosphate (PIP2) to inositol 1,4,5-trisphosphate (IP3) and diacylglycerol (DAG), and is instrumental in epidermal morphogenesis [29], [39]. Pleckstrin homology domain specifically binds to phosphatidylinositol-4, 5-bisphosphate (PI(4,5)P2) and any mutation in this domain puts synaptic vesicle transport into disarray [40].

Many of the EF hand proteins have Receptor-mediated endocytosis (RME) domain which has been shown to extend help in endocytosis process [37]. Domains such as calponin homology and spectrin suggest that the proteins containing them interact with actin and are part of the cytoskeletal proteins [35], [41]. One EF hand protein has BTB Profile [42]. Another EF hand protein has src homology domain which was first identified in oncoproteins [43]. Extra domains in EF-hand proteins might help in evaluating the function of these proteins.

The unique aspect of the results is that the *Caenorhabditis* has so many three- to four-EF-hand-containing proteins with no other domain. These proteins are likely to bind only Ca^+2^ ions, as these do not have any extra sequences corresponding to other domain. These proteins's architecture looks quite similar to CaM. After binding Ca^+2^ these may undergo conformational change which makes them suitable for the activation of associated proteins.

### Expression of EF and non EF-hand proteins with Ca^+2^/metal binding motif

Data presented above states that *Caenorhabditis* has a large number of EF-hand proteins non EF-hand proteins with Ca^+2^/metal binding motif. Are all these proteins functional? It is not possible to say anything about the functionality of a gene just on the basis of computational analysis. However, one of the ways to know the functionality of the genes is to check the expression profile of genes. If gene is expressed at both RNA and protein levels, there is a strong possibility that the gene is functional. Table S1 & S2 also list whether protein is expressed or not. Overall it appears that a majority fraction of identified EF-hand proteins is expressed in *Caenorhabditis*.

### Presence of identical EF-hand proteins in more than one species of *Caenorhabditis*

Presence of these proteins was also checked in other species of *Caenorhabditis*. Some of these were found and summary of the results is shown in Table 4. NCS, CaM, CaM-like protein and Troponin C are present in more than one species of *Caenorhabditis* [12], [32], [34]. Presence of CaM across the species of *Caenorhabditis* confirms the belief that one of the most important EF-hand proteins is CaM. Troponin C is present in *C. remanei, C. brenneri, C. briggsae*. There can be functional similarity in same protein from different species. Presence of identical proteins in more than species also helps in understanding the importance of that protein in the evolution. It also tells how essential that protein is for the functioning of that organism.

### Nearest homologue of EF-hand proteins present in species other than *Caenorhabditis*

Table S3 lists the nearest homologue of EF-hand protein present in species other than *Caenorhabditis*. Table S3 not only gives the name of the closest homologue but also the percentage of identity with homologue. Closest homologue identity ranges from 34% to 98%. Majority of the homologues belong to *Ascaris, Loa, Dugesia, Trichinella, Dictyostelium* and *Onchocerca*.

CaM and CaM related protein homologues share a great deal of identity. Identity reaches as high as 92%. The reason for high percentage could be that CaM is highly conserved protein and very little change happens during the evolution of new species. In most of the cases, the closest homologue is present in worms. It satisfies the fact that on evolutionary ladder worms would be closest neighbour of *Caenorhabditis*. Many of the homologues found out in other organism are putative ones. So some inference about their function could be guessed from function of known homologue partner. Nearest homologue of *C. elegans* hypothetical protein M04F3.4 is *Loa loa* programmed cell death protein-6. Identity between them is around 77%. So the function of *C. elegans* hypothetical protein M04F3.4 could also be speculated on the basis of high degree of identity. In many cases same protein is closest homologue of more than one EF-hand proteins. Only difference among them is percentage of identity. Like *C. briggsae AF16* HP CBG_02410 and *C. brenneri* troponin C closest homologue is *Loa loa* putative uncharacterized protein and percentage of identity is 44% and 95% respectively.

### Phylogenetic analysis of EF-hand containing proteins

Full length EF-hand protein sequences identified by InterProScan were aligned using MAFFT [44]. Some of the sequences which were less than 100 amino acids were not included in phylogenetic analysis. EF-hand sequences which were more than 2200 amino acids long were not included in Phylogenetic analysis. This was done so that large number of gaps is not produced during the alignment because of large disparity in the sequence length.

**Figure 4:**
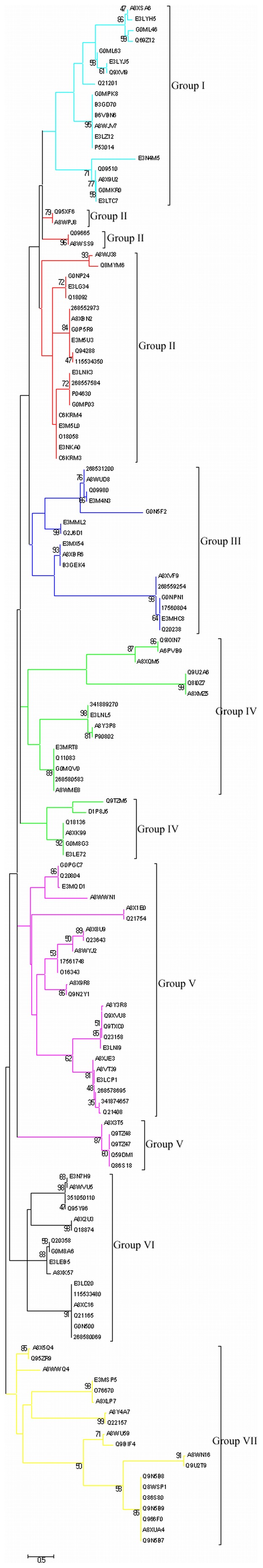
Phylogenetic tree to show the overall relatedness of the EF-hand proteins. All EF-hand proteins were aligned using MAFFT and analyzed using maximum likelihood method in MEGA5. Numbers represent the bootstrap values of 1000 replicates. The expanded groups are shown in Figures 5–11.

**Figure 5:**
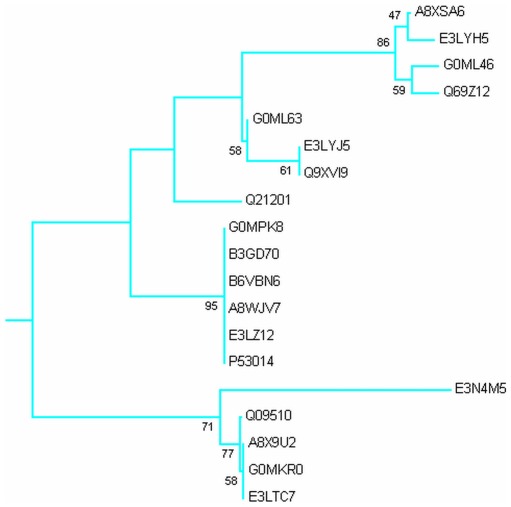
Group I. Tree showing all proteins included in this group.

**Figure 6 pone-0036770-g006:**
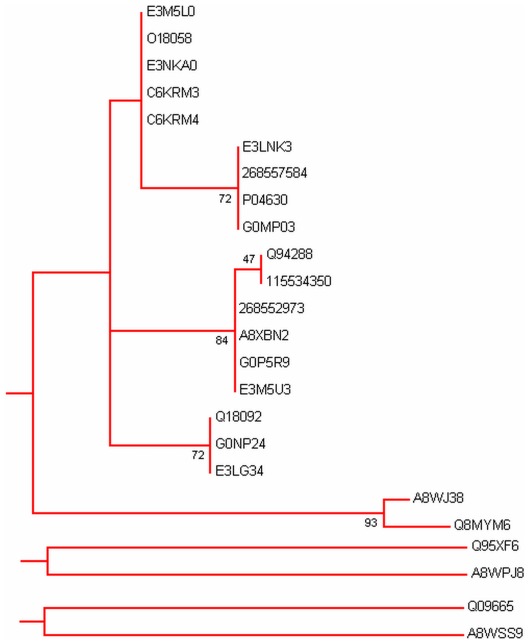
Group II. Tree showing all proteins included in this group.

**Figure 7 pone-0036770-g007:**
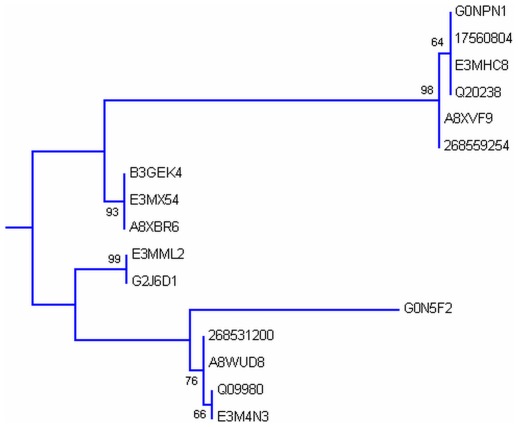
Group III. Tree showing all proteins included in this group.

**Figure 8 pone-0036770-g008:**
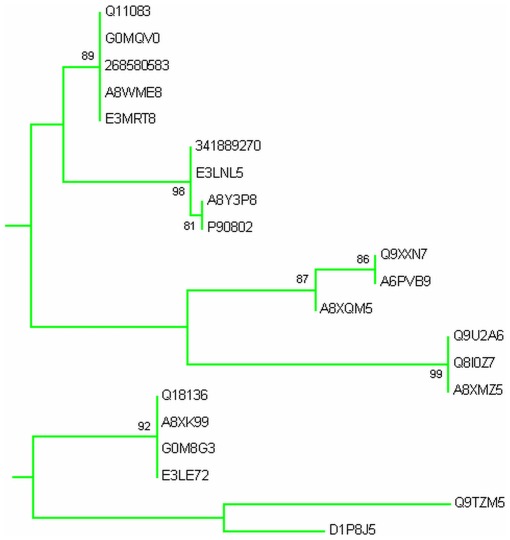
Group IV. Tree showing all proteins included in this group.

**Figure 9 pone-0036770-g009:**
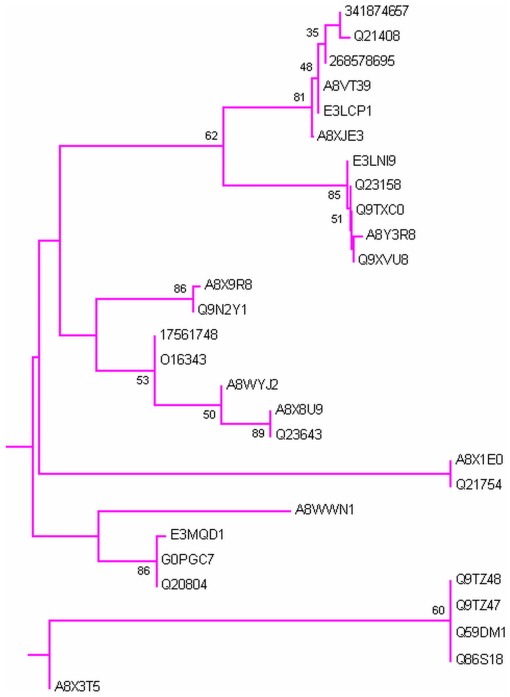
Group V. Tree showing all proteins included in this group.

**Figure 10 pone-0036770-g010:**
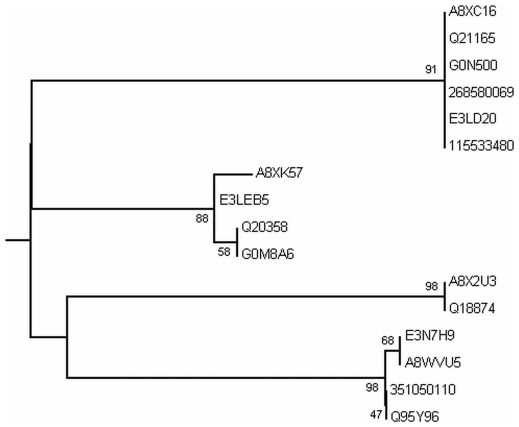
Group VI. Tree showing all proteins included in this group.

**Figure 11:**
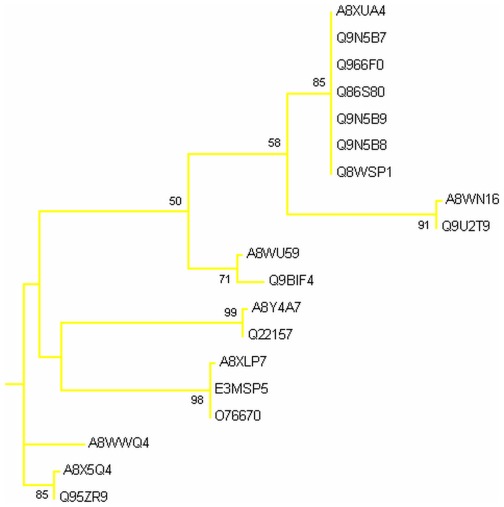
Group VII. Tree showing all proteins included in this group.

Phylogenetic analysis was carried out by MEGA5 using maximum likelihood method. A bootsrap consensus tree was constructed. The tree was used to identify group of proteins and closely related proteins, where total seven groups could be identified. Figure 4 shows the overall tree with members from each group. Figures 5, 6, 7, 8, 9, 10, and 11 are the expanded trees for each group.

#### Group I

In this group there is good number of hypothetical proteins (Figure 5). MLC-3 and MLC-4 [36] have been also grouped along with hypothetical proteins. Not much has been written about these proteins in the literature. Some of them contain domains that give some clue to their function like Myosin Light Chain regulation etc. Most of them have two EF-hands.

#### Group II

Group II (Figure 6) is a collection of CaM and CaM related proteins. CaM [12] is small acidic protein. It is highly conserved protein and has four EF-hands. Four EF-hands exist in two pair and both the pair is connected through central helix [12]. All the four EF-hands bind Ca^+2^ ions and binding of Ca^+2^ to EF-hands bring conformational change in the CaM which then allows CaM to interact with target proteins to modulate their activity or function. More than five CaM related proteins are present in *Caenorhabditis* and almost all of them are highly similar. There is some variation in the length and number of EF-hands of these proteins. CAL-4 has three EF-hands. CAL-1, CAL-2 and CAL-3 have four EF-hands. It also includes Troponin C which too has four EF-hands. As far as expression of these proteins are concerned not much is known except CAL-1 which is expressed in *C. Elegans*. The Ca^+2^ binding ability of these proteins have not been experimentally verified but amino acid sequence composition indicates that these should have strong Ca^+2^ affinity. CaM has also been shown to be involved in the activation of protein kinases [16].

#### Group III & Group VI

Group III includes MLC-2, PAT 10 [32] and hypothetical proteins. PAT 10 (Paralysed arrest at two-fold protein) has been shown to be involved in the embryo & larval development, locomotion, growth, muscle contraction, endocytosis, reproduction, skeletal muscle assembly. PAT 10 [32] has four EF-hands but its Ca^+2^ binding ability has not been proven (Figure 7 & 8). Through electronic annotation we have come to know that PAT 10 has 2 Ca^+2^ binding motifs. MLC-2 two has 2 EF-hands.

Group VI mostly comprises of proteins with four and five EF-hands and most of them are uncharacterized and hypothetical proteins. Other members of Group VI are K03A1.4 and C56A3.6a. C56A3.6a has 3 EF-hands whereas K03A1.4 has four EF-hands. Both of them have no extra domain.

#### Group IV

It contains Vitellogenin linked protein [45] (Figure 9). Vitellogenin [45] is precursor of the egg-yolk proteins that are sources of nutrients during embryonic development. It is major source of energy during embryonic development. Vitellogenin is carrier protein that transports lipid and other things. Btb and math domain containing protein 25 (BATH-25) and CAL-5 are part of this group. BATH-25 has two EF-hands and both EF-hands display Ca^+2^ binding ability. CAL-5 has four EF-hands and all four EF-hands show liking for Ca^+2^. There is also good number of hypothetical and uncharacterized proteins present in this group. Some other members are Y48B6A.6b (two EF-hands), Y48B6A.6c (two EF-hands) and CaBP F21A10.1 (three EF-hands). InterProScan shows that both can bind Ca^+2^.

#### Group V

All cytoskeleton proteins fall in this group (Figure 10). Spectrin1 which binds with actin is in this group [46]. Spectrin provides a scaffold to the actin network. Spectrins are very large protein having length more than 1000–2000 amino acids. Spectrin has two EF-hands. Another member α-actinins which belongs to spectrin gene superfamily is part of the cytoskeleton architecture [47]. Cytoskeleton proteins provide support to plasma membrane and help in the locomotion of the organism. A family of phosphatidylinositol specific PLC [29] is known but only two of them have EF-hand domain. PLC hydrolyzes Phosphatidylinositol-4, 5-bisphosphate and its activity is completely Ca^+2^ dependent. CaBP1 which has 8 EF-hands probably plays a role in the signal transduction. ZK856.8 has three EF-hands and computationally it has been found out that it is involved in the signal trassduction and locomotion. W04D2.1 [47] has calponin homology domain and through mutant phenotype experiments it has been proven that it is involved in the embryo development and mitosis. CNB-1, a four EF-hand protein is involved in movement, fertility, egg laying, and growth in *Caenorhabditis elegans* [48].

#### Group VII

This group at the end of the Phylogenetic tree comprises of NCS-2, NCS-3 and different isoforms of RME. NCS-2 & NCS-3 are members of large family of NCS (Figure 11). Not all the EF-hands of NCS show affinity toward Ca^+2^. RME-1 isoform a, c, e and f have one EF-hand and on the basis of InterProScan we can say that it might bind Ca^+2^ ions. RME has Eps 15 homology domain. From electronic annotation it has been inferred that it has GTPase activity (GTP binding). ITSN-1 [49] and Y116A8C.36a have three domains – two EF-hands, Eps 15 homology and Src homology. EHS 1 has two EF-hands and one Eps 15 homology domains [50]. T04F3.2 not only contains two EF-hands but also one thyroglobulin domain. T04F3.2 also might be involved in the signal transduction. Thyroglobulin is thought to be involved in the control of proteolytic degradation. The domain usually contains six conserved cysteines. Some hypothetical and uncharacterized proteins with six EF-hands are also enclosed in this.

### Prediction of Ca^+2^ binding constants

Ca^+2^ binding attribute of an EF-hand sequence is governed by five amino acids of 12 amino acids Ca^+2^ binding loop sequence of EF-hand. If any mutation occurs in this loop sequence then there is an alteration in the Ca^+2^ binding affinity of the EF-hand. CaM has four EF-hands and all four EF-hands show high degree of Ca^+2^ binding affinity. Keeping this point in view we retrieved more than 10 CaM sequences of 10 different organisms. Ca^+2^ binding loop sequences of all CaMs were aligned and a consensus sequence was generated. More than 40 EF-hand sequences were aligned. This consensus sequence was compared with the Ca^+2^ binding loop sequence of EF-hand proteins and if there was 100% identity with consensus sequence at five critical positions of the loop that might be involved in the Ca^+2^ binding then it was considered as strong Ca^+2^ binding affinity. If the identity at five critical positions of the loop drops down to less than 70% then it was considered medium. And if the identity comes as less as 50% then it was regarded as low. As far as assignment of some number to the strong, medium or low Ca^+2^ binding constant is concerned, it was quiet difficult task in view of the fact that there was some variation among the Ca^+2^ binding constants of different CaM. This variation happens because there is some variation in the sequence also. To sort out the issue we tried to round about range value based on the Ca^+2^ binding constants of some CaM [51], [52] which might give some approximate idea about the Ca^+2^ binding constants. For strong, a range of 10^4^ to 10^7^ was considered; medium, less than 10^4^; low less than 10^2^.

Though there could be lot of debate on the assignment of some numerical value to the strong, medium and low Ca^+2^ binding constants but one thing about this comparison is sure that the loop sequence which is 100% identical with the CaM consensus loop sequence is most probably going to have Ca^+2^ binding constant, comparable to that of CaM. Ca^+2^ binding loop sequence which is less than 100% identical to the CaM consensus loop sequence should have less Ca^+2^ binding constant in comparison to that of CaM. Table S4 lists the Ca^+2^ binding constant of EF-hand CaBP. Here, out of the all studied proteins, Ca^+2^ binding affinity was significant in most of the cases and few were of medium affinity but we could not find any sequence having very low affinity.

## Discussion

CaM-like protein (gi: 37699821) model shares sequence homology of 65% with the template. The sequential and structural information between the CaM-like protein (gi: 37699821) from *C. elegans* and *Drosophila melanogaster* CaM are well conserved. The stable structure is used for docking with IQ1 motif. In this study it was found that A112, A39, E36 of CaM-like protein are important for strong hydrogen binding interaction with IQ1 motif. CaM-like protein residues F165, F169, L29, E33, F44, L57, M61, M96, M97, M108, G65, V115, F93, N104, and E144 play important role in interaction with IQ1 motif.

It is not clear why *Caenorhabditis* requires such a large number of Ca^+2^ sensors, unlike many other organisms? Some of the probable reasons would be that Ca^+2^, being major player in diverse functions and some of these being compartmentalized in different cellular locations, different CaBP may participate in different functions which are spatially and temporally separated. *Caenorhabditis* EF hand proteins control diverse array of cellular functions. MLC 4, Spectrin and alpha actinin are associated with cytoskeleton architecture [35], [36], [47]. PLC is a major player in signal transduction in *Caenorhabditis* [29]. ITSN 1 and EHS 1 are involved in vesicle recycling at the neuromuscular junction [49], [50]. RME-1, a conserved EH-domain protein, functions in endocytic recycling [37]. The wide variety of domains in the EF-hand proteins also shows the diversity of cellular processes in which Ca^+2^ is involved.

Complexity of Ca^+2^ signalling is enhanced due to the existence of many families of proteins. More than one isoform of CaM, NCS and RME are present in *Caenorhabditis*. The regulation of expression and kinetics of interaction of these isoforms with different proteins makes cell signalling processes further complex. The variation in the Ca^+2^ binding affinity adds a new dimension to the regulation of CaBP for fast association and fast dissociation is required for the switching on and off of CaBP. In summary, *Caenorhabditis* exhibits unusual array of Ca^+2^ sensors, indicating a complex network of Ca^+2^ signaling pathways. Further characterization of the CaBP is required for complete understanding of the roles of Ca^+2^ and Ca^+2^ signalling network.

## Methods

### Homology Modelling and Docking

Amino acids sequence of *C. elegans* CaM-like protein (gi: 37699821) was retrieved from Swiss-Prot database [53]. Template was searched by BLAST-P analysis against PDB database. Crystal structure of *Drosophila melanogaster* CaM (2BKH|B) available at PDB was used as template for modelling. Sequence alignment between the model sequence and template was done with ClustalW [54]. Homology model *C. elegans* CaM-like protein was built by Modeller [55] version 9v7. Model was checked using PROCHECK [56] and Prosa-web [57]. Further, models were subjected to energy minimization using GROMOS96 implemented via Swiss-pdb viewer [58] and refinement in COOT [59]. RMSD between template and the model structure was evaluated using the Pymol [60]. Docking of model structure was performed by GRAMM-X [61]. Best docked structure based on lowest energy score was chosen for further analysis. Hydrogen and hydrophobic interactions between protein and ligand were analyzed by PyMOL [60]. Figures representation was generated with PyMOL [60].

### Sequence Analysis

Amino acid sequences of all annotated CaBP and EF-hand proteins were retrieved from *Caenorhabditis* genome database (http://www.ncbi.nlm. nih.gov). Each sequence was used for BLAST sequence (http://www.ncbi.nlm.nih.gov/BLAST) similarity search against *Caenorhabditis* genome database. InterProScan was extensively used to look for the presence of EF-hands and other domain in these proteins [24].

A number of search tools such as reverse BLAST, PSI-BLAST, CD-search, HMM search and other sequence searching algorithm was also used to identify maximum number of EF-hands. Other databases like Pfam (http://www.pfam.wustl.edu/hmmsearch.shtml), UniProt (http://www.uniprot.org) and WORM database were extensively searched for the EF-hand proteins and any other information related to it. Nearest homologues in species other than *Caenorhabditis* were also collected using BLAST.

Full length EF-hand protein sequences identified by InterProScan were aligned using MAFFT v6.707 with FFT-NS-i (slow; iterative refinement method) [44]. Some of the sequences which were less than 100 amino acids were not included in phylogenetic analysis. EF-hand domain sequences which were more than 2200 amino acids long were not included in the Phylogenetic analysis. This was done so that large number of gaps is not produced during the alignment because of large disparity in the sequence length.

### Phylogenetic Analysis

The phylogenetic tree and branch support values were estimated using Maximium Likelihood (ML) methodology of phylogenetic reconstruction using MEGA 5 [62]. ML analyses was carried out with starting from the BIONJ tree, and the gamma distribution for rate heterogeneity across sites (Γ) was modeled with a five-category Γ distribution and a shape parameter equal to 8.2294. The WAG substitution model [63] was selected by MEGA 5, following the Bayesian Information Criterion, as best-fitting model among the models tested, that could be used in ML. Bootstrap values were based on 1000 pseudo-replicates to estimate support for the nodes of the ML tree.

### Prediction of Calcium Binding Constant

For the prediction of Ca^+2^ binding constants, CaM sequences of 10 different organisms were retrieved from swiss prot database. Ca^+2^ binding loop sequences of all CaM were aligned and a consensus sequence was generated. More than 40 EF-hand sequences were aligned. This consensus sequence was compared with the Ca^+2^ binding loop sequences of EF-hand proteins.

## Supporting Information

Table S1 EF-hand containing proteins in *Caenorhabditis*.
(PDF)

Table S2 Non EF-hand with calcium/metal binding motif proteins in *Caenorhabditis*.
(PDF)

Table S3 Nearest homologue of EF-hand Proteins in organism other than *Caenorhabditis* genus.
(PDF)

Table S4 Calcium binding constant of EF-hand CaBP.
(PDF)
