# Correction: *In Silico* Prediction and Analysis of *Caenorhabditis* EF-hand Containing Proteins

**DOI:** 10.1371/annotation/f7f4b3be-c835-440b-a851-7eb62c142260

**Published:** 2012-08-06

**Authors:** Manish Kumar, Shadab Ahmad, Ejaz Ahmad, Muheet Alam Saifi, Rizwan Hasan Khan

The results reported under the 'Prediction of calcium binding constant' section of the article should be disregarded.

A collaborator of the authors wants to further verify the results reported under this section of the article, those results will be reported separately with additional supporting experimental data. Therefore, publication of the said portion of the article with verification will be much more meaningful.

Since the results under the 'Prediction of calcium binding constant' section are not central to the main theme of the current article, the removal of this part of the results does not impact the conclusions reported in this publication.

The parts of the article that should be disregarded are as follows:

Abstract: Last but one sentence.

Results: Prediction of Ca+2 binding constants

Discussion: Lines 6-8 from the last paragraph

Methods: Prediction of Ca+2 binding constants

Supporting Information: Table S4

References: 51 & 52

