# Supplementary material for: In Silico Prediction and Analysis of Caenorhabditis EF-hand Containing Proteins
Source: PLoS One. 2012 May 25;7(5):e36770. doi: 10.1371/journal.pone.0036770 (PMC3360750; doi:10.1371/journal.pone.0036770)
Supplement: Table S1 — EF-hand containing proteins in Caenorhabditis. (PDF) [file pone.0036770.s001.pdf]

Table S1. EF-hand containing proteins in *Caenorhabditis*

UniProtID/gi	Predicted Name	Organism	Length	EF-Hands	Other domain	PE	Molecular Function	Source	Biological function	Source
G0NP24	HP CAEBREN_11606	*C. brenneri*	171	4		P				
E3LG34	CRE-CAL-2	*C. remanei*	171	4		P	CaB	IEA		
E3LNK3	CRE-CAL-1	*C. remanei*	198	4		P	CaB	IEA		
P04630	CAL-1	*C. elegans*	161	4		T	CaB	IEA		
G0MP03	HP CAEBREN_30810	*C. brenneri*	168	4		P				
268557584	CBR-CAL-1	*C. briggsae*	180	4						
268552973	CBR-CAL-3	*C. briggsae*	164	4						
E3M5U3	CRE-CAL-3	*C. remanei*	231	4		P	CaB	IEA		
G0P5R9	HP CAEBREN_12026	*C. brenneri*	181	4		P				
A8XBN2	CBR-CAL-3	*C. briggsae*	233	4		P	CaB	IEA		
E3M5L0	Uncharacterized	*C. remanei*	182	4		P	CaB	IEA		
E3NKA0	HP CRE_15028	*C. remanei*	290	3		P	CaB	IEA		
O18058	CAL-4	*C. elegans*	182	3		P	CaB	IEA		
C6KRM4	T07G12.1b	*C. elegans*	208	3		P	CaB	IEA		
O16305	Calmodulin (CMD-1)	*C. elegans*	149	4		T	CaB, protein binding	IEA, IPI	apoptosis, reproduction	IMP
C6KRM3	T07G12.1a	*C. elegans*	236	3		P	CaB	IEA		
Q94288	Calmodulin related genes protein 3	*C. elegans*	167	4		T	CaB	IEA		
115534350	CAL-3	*C. elegans*	234	4						
341889270	HP CAEBREN_06721	*C. brenneri*	201	2						
E3LNL5	HP CRE_27132	*C. remanei*	210	4		P	CaB	IEA		
P90802	HP E02A10.3	*C. elegans*	202	4		T	CaB	IEA		
A8Y3P8	HP CBG23534	*C. briggsae*	201	4		P	CaB	IEA		
Q09665	Troponin C, isoform 2 (TNC-2)	*C. elegans*	160	4		T	CaB	IEA	reproduction, larval development, growth	IMP
A8WSS9	CBR-TNC-2	*C. briggsae*	160	4		P	CaB	IEA		
268532318	CBR-TNC-2	*C. briggsae*	159	4						

Q11083	HP B0563.7	*C. elegans*	229	4		T	CaB	IEA		
Q95XF6	HP Y73B3A.12	*C. elegans*	116	4		P	CaB	IEA	apoptosis, endocytosis, reproduction, cell migration, embryo development, growth, mitosis	IMP
E3MRT8	HP CRE_14767	*C. remanei*	230	4		P	CaB			
268580583	HP CBG00175	*C. briggsae*	230	4						
268531200	HP CBG02410	*C. briggsae*	145	4						
Q09980	HP F12A10.5	*C. elegans*	145	4		T	CaB	IEA		
E3M4N3	HP CRE_11951	*C. remanei*	145	4		P	CaB	IEA		
A8WME8	HP CBG_00175	*C. briggsae AF16*	172	4		P	CaB	IEA		
A8WUD8	HP CBG_02410	*C. briggsae AF16*	150	4		P	CaB	IEA		
B3GEK4	Troponin C	*C. brenneri*	150	4		T	CaB	IEA		
E3MX54	CRE-PAT-10	*C. remanei*	200	4		P	CaB	IEA		
A8XC16	HP CBG_11013	*C. briggsae AF16*	201	4		P	CaB	IEA		
Q18136	Vitellogenin-linked	*C. elegans*	156	4		T	CaB	IEA		
G0M8G3	CBN-CAL-5	*C. brenneri*	156	4		P	CaB	IEA		
G0N500	HP CAEBREN_26130	*C. brenneri*	164	4		P				
E3LE72	CRE-CAL-5	*C. remanei*	156	4		P	CaB	IEA		
Q21165	K03A1.4 a	*C. elegans*	184	4		P	CaB	IEA		
A8XK99	CBR-UVT-2	*C. briggsae*	156	4		P	CaB	IEA		
E3LD20	HP CRE_00078	*C. remanei*	199	4		P	CaB	IEA		
115533480	HP K03A1.4	*C. elegans*	264	4						
G0MQV0	HP CAEBREN_16765	*C. brenneri*	181	3		P				
Q09510	MLC-4	*C. elegans*	172	3		T	CaB	IEA	protein localization, meiosis, mitosis, embryo & larval development, morphogenesis, cell polarity	IMP
A8X9U2	CBR-MLC-4	*C. briggsae*	172	1		P	CaB	IEA		
G0MKR0	HP CAEBREN_07889	*C. brenneri*	172	1		P				
E3LYJ5	HP CBG18252	*C. remanei*	142	2		P	CaB	IEA		

Q9XVI9	HP T12D8.6	*C. elegans*	142	2		T	CaB	IEA		
G0PGC7	HP CAEBREN_10686	*C. brenneri*	163	4		P				
Q20804	cnb-1	*C. elegans*	171	4		T	CaB	IDA		
E3MQD1	CRE-CNB-1	*C. remanei*	171	4		P	CaB	IEA		
E3LTC7	CRE-MLC-4	*C. remanei*	188	1		P	CaB	IEA		
Q21201	HP K04C1.4	*C. elegans*	143	3		T	CaB	IEA		
G0ML63	HP CAEBREN_02413	*C. brenneri*	156	3		P				
A8XK57	HP CBG14543	*C. briggsae*	159	4		P	CaB	IEA		
B6VBN6	HP Cbre_JD16.001	*C. brenneri*	152	2		P	CaB	IEA		
G0MPK8	CBN-MLC-3	*C. brenneri*	152	2		P				
E3LZ12	CRE-MLC-3 protein	*C. remanei*	153	2		P	CaB	IEA		
B3GD70	MLC-3	*C. brenneri*	152	2		T	CaB	IEA		
A8WJV7	CBR-MLC-3	*C. briggsae*	153	2		P	CaB	IEA		
P53014	MLC-3	*C. elegans*	153	2		T	CaB	IEA	locomotion, oviposition	IMP
A8XVF9	HP CBG_19361	*C. briggsae AF16*	179	1		P	CaB	IEA		
Q20358	HP F43C9.2	*C. elegans*	159	4		T	CaB	IEA		
G0M8A6	HP CAEBREN_15606	*C. brenneri*	159	4		P				
E3LEB5	HP CRE_00789	*C. remanei*	159	4		P	CaB	IEA		
268559254	HP CBG19361	*C. briggsae*	137	2						
G0NPN1	HP CAEBREN_20342	*C. brenneri*	179	1		P				
E3MHC8	HP CRE_22883	*C. remanei*	179	1		P	CaB	IEA		
A8XSA6	HP CBG18268	*C. briggsae*	147	2		P	CaB	IEA		
E3LYH5	HP CRE_04650	*C. remanei*	153	2		P	CaB	IEA		
G0ML46	HP CAEBREN_22778	*C. brenneri*	153	2		P				
Q20238	F40F9.8	*C. elegans*	179	1		P	CaB	IEA		
Q69Z12	HP K08E3.10	*C. elegans*	153	2		P	CaB	IEA		
17560804	HP F40F9.8	*C. elegans*	137	1						
E3N7H9	HP CRE_13655	*C. remanei*	172	5		P	CaB	IEA		
Q95Y96	HP M04F3.4	*C. elegans*	157	4		T	CaB	IEA		
351050110	M04F3.4 a	*C. elegans*	172	5						

17561748	HP F59D6.7	*C. elegans*	213	4						
Q9TZM5	bath-25	*C. elegans*	364	2	BTB	P	CaB	IEA		
O16343	F59D6.7	*C. elegans*	195	4		P	CaB	IEA		
A8XLP7	HP CBG15199	*C. briggsae*	233	5		P	CaB	IEA	ST	IEA
E3MSP5	HP CRE_16173	*C. remanei*	235	6		P	CaB	IEA	ST	IEA
A8WVU5	HP CBG03955	*C. briggsae*	173	5		P	CaB	IEA		
E3N4M5	HP CRE_29149	*C. remanei*	186	2		P	CaB	IEA		
G0N5F2	HP CAEBREN_02194	*C. brenneri*	189	4		P				
A8VT39	Spectrin	*C. remanei*	171	2		T	CaB	IEA		
E3MML2	HP CRE_03312	*C. remanei*	170	2		P	CaB	IEA		
Q21408	spc-1	*C. elegans*	2427	2	SH3	T	CaB	IEA	Larval development, morphogenesis, growth, locomotion, mitosis	IMP
E3LCP1	CRE-SPC-1	*C. remanei*	2370	2	SH3	P	CaB	IEA		
E3LNI9	CRE-ATN-1	*C. remanei*	921	2	CH	P	CaB, actin binding	IEA		
268578695	CBR-SPC-1	*C. briggsae*	2427	2	SH3					
G2J6D1	CBR-MLC-2.2	*C. briggsae*	170	2		P				
341874657	CBN-SPC-1	*C. brenneri*	2427	2	SH3					
A8WWQ4	CBR-NCS-2	*C. briggsae*	190	3		P	CaB	IEA		
A8Y4I1	CBR-CNB-1	*C. briggsae*	171	4		P	CaB	IEA		
A8X5Q4	CBR-NCS-3	*C. briggsae*	174	2		P	CaB	IEA		
A8X9R8	Uncharacterized	*C. briggsae*	196	2		P	CaB	IEA		
A8XBR6	CBR-PAT-10	*C. briggsae*	161	4		P	CaB	IEA		
A8WPJ8	CBR-CMD-1	*C. briggsae*	149	4		P	CaB	IEA		
A8XQM5	Uncharacterized	*C. briggsae*	224	3		P	CaB	IEA		
A8WYJ2	Uncharacterized	*C. briggsae*	199	3		P	CaB	IEA		
A8X2U3	Uncharacterized	*C. briggsae*	456	3		P	CaB	IEA		
A8XEQ0	Uncharacterized	*C. briggsae*	81	4		P	CaB	IEA		
B3GDA0	Troponin C	*C. brenneri*	161	4		T	CaB	IEA		
B3GD87	Troponin C	*C. brenneri*	37	1		T	CaB	IEA		
D1P8J5	Uncharacterized	*C. elegans*	176	2		P	CaB	IEA		
Q9N2Y1	Uncharacterized	*C. elegans*	196	2		T	CaB	IEA	larval development, growth	IMP

Q9XXN7	F21A10.1a	*C. elegans*	224	3		T	CaB	IEA		
Q9U2A6	Y48B6A.6a	*C. elegans*	687	2		T	CaB	IEA		
Q3V5K4	Uncharacterized	*C. elegans*	97	2		P	CaB	IEA		
A8XMZ5	Y48B6A.6c	*C. elegans*	179	2		P	CaB	IEA		
Q8I0Z7	Y48B6A.6b	*C. elegans*	203	2		T	CaB	IEA		
Q2L6W4	Uncharacterized	*C. elegans*	43	1		T	CaB	IEA		
Q18874	C56A3.6a	*C. elegans*	477	3		T	CaB	IEA		
Q95ZR9	calcium sensor 3	*C. elegans*	199	4		T	CaB	IEA		
P91423	Uncharacterized	*C. elegans*	81	2		T	CaB	IEA		
Q8MYM6	Y39B6A.38	*C. briggsae*	410	1	EH	T	CaB	IEA		
Q9BIF4	EHS-1	*C. briggsae*	796	2	EH	T	CaB	IEA		
Q18092	C18E9.1	*C. elegans*	171	4		T	CaB	IEA	embryo development	IMP
P91328	Troponin C or PAT 10	*C. elegans*	161	4		T	CaB	IEA	Embryo/larval development, locomotion, growth, muscle contraction, endocytosis, reproduction, skeletal muscle assembly	IMP
A6PVB9	F21A10.1b	*C. elegans*	145	3		P	CaB	IEA		
O76670	Uncharacterized		236	6		T	CaB	IEA	ST	IEA
P36609	NCS-2		190	3		T	CaB	IEA		
A8Y3R8	CBR-ATN-1	*C. briggsae*	894	2	CH	P	CaB, actin binding	IEA		
A8XJE3	CBR-SPC-1	*C. briggsae*	2467	2	SH3	P	CaB	IEA		
Q9TXC0	Alpha-ACTININ	*C. elegans*	910	2	CH	T	CaB, actin binding	IEA		
Q9XVU8	W04D2.1	*C. elegans*	894	2	CH	T	CaB, actin binding	IEA		
Q23158	W04D2.1a	*C. elegans*	920	2	CH	T	CaB,actin binding	IEA	embryo development, mitosis	IMP
A8X1E0	CBR-PLC-4	*C. briggsae*	752	2	PH, X, Y, C2	P	CaB, actin binding, PLC	IEA	ST, lipid metabolic	IEA
Q21754	PLC4	*C. elegans*	751	2	X, Y, C2, PH	T	CaB, actin binding, PLC	IEA	ST, lipid metabolic	IEA
A8Y4A7	Uncharacterized	*C. briggsae*	261	1	Thr	P	CaB	IEA	ST	IEA
A8WWN1	Uncharacterized	*C. briggsae*	143	3		P	CaB	IEA	ST	IEA
A8X8U9	Uncharacterized	*C. briggsae*	195	3		P	CaB	IEA	ST	IEA
A8X3T5	CBR-CBN-1	*C. briggsae*	710	8		P	CaB	IEA	ST	IEA
Q9TZ48	CaBP1	*C. elegans*	588	8		T	CaB	IEA	ST	IEA

Q22157	T04F3.2	*C. elegans*	260	2	Thr	T	CaB	IEA	ST	IEA
Q9TZ47	CaBP1	*C. elegans*	482	8		T	CaB	IEA	ST	IEA
Q86S18	CaBP1	*C. elegans*	462	8		T	CaB	IEA	ST	IEA
Q23643	ZK856.8	*C. elegans*	195	3		T	CaB	IEA	locomotion, ST	IEA
Q59DM1	CaBP1	*C. elegans*	679	8		T	CaB	IEA	ST	IEA
A8WU59	CBR-EHS-1	*C. briggsae*	794	2	EH	P	CaB	IEA		
A8WJ38	CBR-REPS-1	*C. briggsae*	252	1	EH	P	CaB	IEA		
A8XUA4	CBR-RME-1	*C. briggsae*	611	1	EH	P	GTP binding,CaB, GTPase activity	IEA		
A8WN16	CBR-ITSN-1	*C. briggsae*	1099	2	EH, SH3	P	CaB	IEA		
Q9N5B7	RME-1, isoform a	*C. briggsae*	786	1	EH	T	receptor, CaB, GTPase activity, GTP binding	IEA		
Q8WSP1	RME-1, isoform e	*C. briggsae*	613	1	EH	T	receptor, CaB, GTPase activity, GTP binding	IEA		
Q86S80	RME-1, isoform f	*C. briggsae*	555	1	EH	T	receptor, CaB, GTPase activity, GTP binding	IEA		
Q9N5B9	RME-1, isoform c	*C. briggsae*	589	1	EH	T	receptor, CaB, GTPase activity, GTP binding	IEA		
Q9N5B8	RME-1, isoform b	*C. briggsae*	835	1	EH	T	receptor, CaB, GTPase activity, GTP binding	IEA	embryo development, endocytic recycling, meiosis, endocytosis	IMP

BTB Profile- Broad-Complex, Tramtrack and Bric a brac; SH2- Src homology 2; IMP- Inferred from Mutant Phenotype; CH- Calponin homology; SH3- Src homology3; IEA- Inferred from Electronic Annotation; EH- Eps15 Homology; PH- Pleckstrin homology; X- Phatidylinositol-specific phospholipase X; HP- Hypothetical Protein; Y- Phosphatidylinositol-specific phospholipase Y; Thr- Thyroglobulin; CaB- Calcium Binding; PLC- Phospholipase C; ST- Signal transduction; P- Predicted; T- Transcript; RME – Receptor Mediated Endocytosis; CaBP- Calcium Binding Protein; IPI- Inferred from Physical Interaction; PE- Protein Existence; PAT 10 - Paralysed arrest at two-fold protein 10; NCS - Neuronal Calcium Sensor, MLC- Myosin Light chain; SPC- Spectrin; EF hand no.– Number of EF hands; Length- Number of amino acids; Biological function information was compiled from Uniprot and WORMBASE; All the functions of a protein deduced through one common technique have been put in a one row of the respective protein. If the technique differed for the particular function of the protein then it is kept in the next row of the respective protein.
