# Supplementary material for: In Silico Prediction and Analysis of Caenorhabditis EF-hand Containing Proteins
Source: PLoS One. 2012 May 25;7(5):e36770. doi: 10.1371/journal.pone.0036770 (PMC3360750; doi:10.1371/journal.pone.0036770)
Supplement: Table S2 — Non EF-hand with calcium/metal binding motif proteins in Caenorhabditis. (PDF) [file pone.0036770.s002.pdf]

**Table S2.** Non EF-hand with calcium/metal binding motif proteins in *Caenorhabditis*

| UniProtID/gi | Predicted Name | Organism | Length | Other domain | PE | Molecular Function | Source | Biological function | Source |
|---|---|---|---|---|---|---|---|---|---|
| A8Y1Z6 | CBR-DYB-1 | *C. briggsae* | 622 | zf_zz | P | CaB, ZnB | IEA | | |
| A8XM20 | Uncharacterized | *C. briggsae* | 1276 | ww, zz_zf, zz_ c2h2 | P | ZnB | IEA | | |
| Q9Y048 | Dystrobrevin-1 | *C. elegans* | 590 | zf_zz, zz_zf | T | cytoplasm | IEA | synaptic specificity | IMP |
| | | | | | | glycoprotein | IDA | locomotion, muscle homeostasis, synaptic transmission, sarcomere organization | IMP, IGI |
| Q9TW65 | Dystrophin-1 | *C. elegans* | 3674 | calponin homology | T | transporter | IMP | locomotory behaviour, synaptic transmission | IMP |
| | | | | | | actin binding, ZnB | IEA | homeostasis | IMP |
| | | | | | | | | sarcomere organization | IGI |
| A8XMK5 | CBR-PLL-1 | *C. briggsae* | 1083 | X, Y, C2 | P | CaB, PLC, signal transducer | IEA | ST, metabolic | IEA |
| A8XRA5 | CBR-PLC-1 | *C. briggsae* | 1917 | R, X, Y, C2, RA | P | CaB, PLC, signal transducer, exchange factor | IEA | metabolic, ST | IEA |
| A8XTZ1 | CBR-PLC-3 | *C. briggsae* | 1308 | X, SH2, SH3, PH, Y, C2 | P | PLC, signal transducer | IEA | ST, phospholipid catabolic | IEA |
| A8X232 | CBR-EGL-8 | *C. briggsae* | 1528 | X, Y, C2 | P | PLC, CaB, signal transducer | IEA | ST, lipid catabolic | IEA |

| | | | | | | | | | |
|---|---|---|---|---|---|---|---|---|---|
| Q9U6B9 | PLC beta | *C. elegans* | 1418 | X, Y, C2 | T | PLC, CaB, signal transducer | IEA | ST, lipid catabolic | IEA |
| Q8IA75 | PLC | *C. elegans* | 1075 | X, Y, C2 | T | PLC, CaB, signal transducer | IEA | ST, lipid catabolic | IEA |
| | | | | | | | | pronuclear migration | IMP |
| Q95X30 | Egg laying defective protein-8 | *C. elegans* | 1491 | X, Y, C2 | T | CaB, PLC, signal transducer | IEA | ST, lipid metabolic | IEA |
| Q22070 | T01E8.3 | *C. elegans* | 1350 | X, Y, C2 , PH, SH3, SH2 | T | PLC | IEA | ST, phospholipid catabolic | IEA |
| | | | | | | signal transducer | IEA | endocytosis, reproduction | IMP |
| Q95X29 | Egg laying defective protein-8 | *C. elegans* | 1431 | X, Y, C2 | T | PLC, CaB | IEA | signaling pathway, embryo development, insemination, growth & locomotion regulation, oviposition regulation, oviposition regulation, spicule insertion | IMP |
| | | | | | | signal transducer | IEA | ST, lipid metabolic | IEA |
| Q9TVI2 | PLC210 | *C. elegans* | 1898 | X, Y, C2 , RA, R | T | signal transducer | IEA | lipid metabolic, ST | IEA |
| | | | | | | CaB, PLC, exchange factor | IEA | reproduction | IMP |
| Q7JM51 | F31B12.1b | *C. elegans* | 1868 | X, Y, C2 , RA, R | T | signal transducer, CaB, PLC, exchange factor | IEA | lipid metabolic, ST | IEA |

| | | | | | | | | | |
|---|---|---|---|---|---|---|---|---|---|
| Q9XWB7 | Y75B12B.6 | *C. elegans* | 896 | X, Y | T | CaB, PLC | IEA | lipid metabolic, ST | IEA |
| B3GWB0 | F31B12.1f | *C. elegans* | 1886 | X, Y, C2 , RA, R | P | signal transducer, CaB, PLC, exchange factor | IEA | lipid metabolic, ST | IEA |
| B2D6L1 | F31B12.1d | *C. elegans* | 1875 | X, Y, C2 , RA, R | P | signal transducer, CaB, PLC, exchange factor | IEA | lipid metabolic, ST | IEA |
| Q8IA76 | PLC like protein 1 | *C. elegans* | 1068 | X, Y, C2 | T | signal transducer, CaB, PLC | IEA | ST, lipid metabolic | IEA |
| B2G4Y4 | Phosphoinositide PLC-epsilon | *C. elegans* | 1878 | X, Y, C2 , RA, R | T | signal transducer, CaB, PLC, exchange factor | IEA | lipid metabolic, ST | IEA |
| Q6EZG7 | Egg laying defective protein-8 | *C. elegans* | 592 | X | T | CaB, PLC | IEA | ST, lipid metabolic | IEA |
| A8Y2J4 | CBR-OST-1 | *C. briggsae* | 264 | KAZAL, Ost | P | CaB | IEA | ST | IEA |
| A8XBD1 | Uncharacterized | *C. briggsae* | 275 | Thr | P | CaB | IEA | ST | IEA |
| Q86GV1 | Uncharacterized | *C. elegans* | 234 | Thr | T | CaB | IEA | ST | IEA |
| O44466 | Uncharacterized | *C. elegans* | 226 | Thr | T | CaB | IEA | ST | IEA |
| P34714 | SPARC | *C. elegans* | 264 | KAZAL, Ost | T | CaB | IEA | ST | IEA |
| | | | | | | | | reproduction, growth regulation, embryo & larval development | IMP |
| Q4TTR2 | Osteonectin | *C. remanei (C.* | 106 | | T | CaB | IEA | | |

| | | | | | | | | | |
|---|---|---|---|---|---|---|---|---|---|
| | | *vulgaris)* | | | | | | | |
| O02159 | HP T09B4.4 | *C. elegans* | 142 | | T | CaB | IEA | | |

SH2- Src homology 2; IMP- Inferred from Mutant Phenotype; SH3- Src homology 3; IGI-Inferred from genetic interaction; PH- Pleckstrin homology; IEA- Inferred from Electronic Annotation; RA- Ras-associating; zf_zz- Zinc finger, ZZ type; zz_ c2h2- Zinc finger, C2H2 type; X- Phosphatidylinositol- specific phospholipase X; Y- Phosphatidylinositol-specific phospholipase Y; R-Ras guanine-nucleotide exchange factors catalytic; Thr- Thyroglobulin; CaB- Calcium Binding; PLC- Phospholipase C; ZnB- zinc Binding; ST- Signal transduction; P- Predicted; T-Transcript; IDA -Inferred from Direct Assay; KAZAL- Kazal domain profile; Ost- Osteonectin domain; UniProtID/gi- UniProtID number/gi number. All the functions of a protein deduced through one common technique have been put in a one row of the respective protein. If the technique differed for the particular function of the protein then it is kept in the next row of the respective protein.
