# Supplementary material for: In Silico Prediction and Analysis of Caenorhabditis EF-hand Containing Proteins
Source: PLoS One. 2012 May 25;7(5):e36770. doi: 10.1371/journal.pone.0036770 (PMC3360750; doi:10.1371/journal.pone.0036770)
Supplement: Table S3 — Nearest homologue of EF-hand Proteins in organism other than Caenorhabditis genus. (PDF) [file pone.0036770.s003.pdf]

Table S3. Nearest homologue of EF-hand Proteins in organism other than *Caenorhabditis* genus

Organism	Predicted Name	Length	Uniprot id/gi	Homologue Source	Homologous Protein	Homologous Protein uniprot id	Homologous Protein length	% Identity
C. elegans	C18E9.1	171	Q18092	*Ascaris suum*	CaM-like protein	F1L911	169	78%
C. brenneri	HP CAEBREN_11606	171	G0NP24	*Ascaris suum*	CaM-like protein	F1L911	169	79%
C. remanei	CBR-CAL-2	171	E3LG34	*Ascaris suum*	CaM-like protein	F1L911	169	78%
C. remanei	CRE-CAL-1	198	E3LNK3	*Loa loa*	CaM-like protein	E1FVS4	167	92%
C. elegans	CAL-1	161	P04630	*Loa loa*	CaM-like protein	E1FVS4	167	92%
C. brenneri	HP CAEBREN_30810	168	G0MP03	*Loa loa*	CaM-like protein	E1FVS4	167	89%
C. briggsae	CBR-CAL-1	180	268557584	*Loa loa*	CaM-like protein	E1FVS7	167	89%
C. briggsae	CBR-CAL-3	164	268552973	*Ascaris suum*	CaM-like protein	F1LF35	176	71%
C. remanei	CRE-CAL-3	231	E3M5U3	*Ascaris suum*	CaM-like protein	F1LF35	176	71%
C. brenneri	HP CAEBREN_12026	181	G0P5R9	*Ascaris suum*	CaM-like protein	F1LF35	176	71%
C. briggsae	CBR-CAL-3	233	A8XBN2	*Ascaris suum*	CaM-like protein	F1LF35	176	71%
C. remanei	Uncharacterized	182	E3M5L0	*Loa loa*	Putative uncharacterized protein	E1G7X3	299	80%
C. remanei	HP CRE_15028	290	E3NKA0	*Loa loa*	Putative uncharacterized protein	E1G7X3	299	74%
C. elegans	CAL-4	182	O18058	*Loa loa*	Uncharacterized protein	E1G7X3	299	81%
C. elegans	T07G12.1b	208	C6KRM4	*Loa loa*	Putative uncharacterized protein	E1G7X3	299	75%
C. elegans	T07G12.1a	236	C6KRM3	*Loa loa*	Putative uncharacterized protein	E1G7X3	299	74%
C. elegans	CaM-related genes protein-3	167	Q94288	*Ascaris suum*	CaM-like protein	F1LF35	176	71%
C. elegans	CAL-3	234	115534350	*Ascaris suum*	CaM-like protein	F1LF35	176	71%
C. brenneri	HP CAEBREN_06721	201	341889270	*Dictyocaulus viviparus*	Putative CaM isoform a	Q6QGZ0	210	82%
C. remanei	HP CRE_27132	201	E3LNL5	*Dictyocaulus viviparus*	Putative CaM isoform a	Q6QGZ1	210	82%
C. elegans	HP E02A10.3	202	P90802	*Dictyocaulus viviparus*	Putative CaM	Q6QGZ1	210	82%

					isoform a			
C. briggsae	HP CBG23534	201	A8Y3P8	*D. viviparus*	CaM isoform a	Q6QGZ1	210	82%
C. elegans	tnc-2	160	Q09665	*Ascaris suum*	Troponin C isoform 2	F1LF05	162	89%
C. briggsae	CBR-TNC-2	160	A8WSS9	*Ascaris suum*	Troponin C isoform 2	F1LF05	162	90%
C. elegans	HP B0563.7	229	Q11083	*Ascaris suum*	CaBP	F1LB89	216	47%
C. elegans	HP Y73B3A.12	116	Q95XF6	*Dugesia japonica*	CaM	Q5H765	149	79%
C. remanei	HP CRE_14767	230	E3MRT8	*Ascaris suum*	CaBP	F1LB89	216	43%
C. briggsae	HP CBG00175	230	268580583	*Ascaris suum*	CaBP	F1LB89	216	46%
C. briggsae	HP CBG02410	145	268531200	*Trichinella spiralis*	EF hand domain containing protein	E5SQ18	239	46%
C. elegans	HP F12A10.5	145	Q09980	*Trichinella spiralis*	EF hand domain containing protein	E5SQ18	239	46%
C. remanei	HP CRE_11951	145	E3M4N3	*Monodelphis domestica*	Putative uncha-racterized protein	F7D7Y	149	45%
C. briggsae AF16	HP CBG_00175	172	A8WME8	*Ascaris suum*	CaBP	F1LB89	216	45%
C. briggsae AF16	HP CBG_02410	150	A8WUD8	*Loa loa*	Putative uncha-racterized protein	E1FKG3	154	44%
C. brenneri	Troponin C	150	B3GEK4	*Loa loa*	Putative uncha-racterized protein	E1FUX2	163	94%
C. remanei	CRE-PAT-10	200	E3MX54	*Onchocerca volvulus*	EF-hand family protein	C7DRP1	161	93%
C. briggsae AF16	HP CBG_11013	201	A8XC16	*Onchocerca volvulus*	EF-hand family protein	C7DRP1	161	93%
C. elegans	Vitellogenin-linked	156	Q18136	*Angiostrongylus cantonensis*	Putative Vitellogenin-linked transcript family member	C7BVL8	159	83%

C. brenneri	CBN-CAL-5	156	G0M8G3	*Angiostrongylus cantonensis*	Putative Vitellogenin-linked transcript family member	C7BVL8	159	83%
C. brenneri	HP CAEBREN_26130	164	G0N500	*Ascaris suum*	CaM	F1LFS6	147	63%
C. remanei	CRE-CAL-5	156	E3LE72	*Angiostrongylus cantonensis*	Putative Vitellogenin-linked Transcript family member	C7BVL8	159	84%
C. elegans	K03A1.4 a	184	Q21165	*Ascaris suum*	CaM	F1LFS6	147	63%
C. briggsae	CBR-UVT-2	156	A8XK99	*Angiostrongylus cantonensis*	Putative Vitellogenin-linked transcript family member	C7BVL8	159	84%
C. remanei	HP CRE_00078	199	E3LD20	*Ascaris suum*	CaM	F1LFS6	147	63%
C. briggsae	HP CBG11013	243	268580069	*Ascaris suum*	CaM	F1LFS6	147	63%
C. elegans	HP K03A1.4	264	115533480	*Ascaris suum*	CaM	F1LFS6	147	61%
C. brenneri	HP CAEBREN_16765	181	G0MQV0	*Ascaris suum*	CaM-like protein	F1LF35	176	43%
C. elegans	mlc-4	172	Q09510	*Ascaris suum*	Myosin regulatory light chain	F1KZM0	171	85%
C. briggsae	CBR-MLC-4	172	A8X9U2	*Ascaris suum*	Myosin regulatory light chain	F1KZM0	171	87%
C. brenneri	HP CAEBREN_07889	172	G0MKR0	*Ascaris suum*	Myosin regulatory light chain	F1KZM0	171	87%
C. remanei	HP CBG18252	142	E3LYJ5	*Loa loa*	Myosin	E1G510	147	78%
C. elegans	HP T12D8.6	142	Q9XVI9	*Loa loa*	Myosin	E1G510	147	78%
C. brenneri	HP CAEBREN_10686	163	G0PGC7	*Loa loa*	Protein phosphatise-3	E1FR52	170	92%

C. elegans	cnb-1	171	Q20804	*Ascaris suum*	Calcineurin subunit B type 2	F1L7X3	171	90%
C. remanei	CRE-CNB-1	171	E3MQD1	*Ascaris suum*	Calcineurin subunit B type 2	F1L7X3	171	91%
C. remanei	CRE-MLC-4	188	E3LTC7	*Ascaris suum*	Myosin regulatory light	F1KZM0	171	79%
C. elegans	HP K04C1.4	143	Q21201	*Toxocara canis*	TcH500 protein	P91814	148	49%
C. brenneri	HP CAEBREN_02413	156	G0ML63	*Loa loa*	Myosin	E1G510	147	71%
C. briggsae	HP CBG14543	159	A8XK57	*Ascaris suum*	CaM	F1LDU1	119	76%
C. brenneri	HP Cbre_JD16.001	152	B6VBN6	*Setaria digitata*	Alkali MLC	C7DXW9	149	84%
C. brenneri	CBN-MLC-3	152	G0MPK8	*Setaria digitata*	Alkali MLC	C7DXW9	149	84%
C. remanei	CRE-MLC-3 protein	153	E3LZ12	*Setaria digitata*	Alkali MLC	C7DXW9	149	84%
C. brenneri	MLC-3	152	B3GD70	*Setaria digitata*	Alkali MLC	C7DXW9	149	84%
C. briggsae	CBR-MLC-3	153	A8WJV7	*Setaria digitata*	Alkali MLC	C7DXW9	149	84%
C. elegans	MLC-3	153	P53014	*Setaria digitata*	Alkali MLC	C7DXW9	149	81%
C. briggsae AF16	HP CBG_19361	179	A8XVF9	*Dictyostelium discoideum*	CaM	P02599	152	34%
C. elegans	HP F43C9.2	159	Q20358	*Ascaris suum*	CaM	F1LDU1	119	75%
C. brenneri	HP CAEBREN_15606	159	G0M8A6	*Ascaris suum*	CaM	F1LDU1	119	76%
C. remanei	HP CRE_00789	159	E3LEB5	*Ascaris suum*	CaM	F1LDU1	119	76%
C. briggsae	HP CBG19361	137	268559254	*Dictyostelium discoideum*	CaM	P02599	152	34%
C. brenneri	HP CAEBREN_20342	179	G0NPN1	*Dictyostelium discoideum*	CaM	P02599	152	34%
C. remanei	HP CRE_22883	179	E3MHC8	*Dictyostelium discoideum*	CaM	P02599	152	32%
C. briggsae	HP CBG18268	147	A8XSA6	*Brugia malayi*	Myosin putative	A8PB51	151	42%
C. remanei	HP CRE_04650	153	E3LYH5	*Caligus rogercresseyi*	Myosin-2 essential light chain	C1BRM7	150	43%
C. brenneri	HP CAEBREN_22778	153	G0ML46	*Brugia malayi*	Myosin putative	A8PB51	151	40%
C. elegans	F40F9.8	179	Q20238	*Dictyostelium discoideum*	CaM	P02599	152	32%
C. elegans	HP K08E3.10	153	Q69Z12	*Ascaris suum*	Myosin-2	F1LFN9	149	40%

					essential light chain			
C. elegans	HP F40F9.8	137	17560804	*Dictyostelium discoideum*	CaM	P02599	152	33%
C. remanei	HP CRE_13655	172	E3N7H9	*Loa loa*	Programmed cell death protein 6	E1GGF7	172	76%
C. elegans	HP M04F3.4	157	Q95Y96	*Loa loa*	Programmed cell death protein 6	E1GGF7	172	76%
C. elegans	M04F3.4 a	172	351050110	*Loa loa*	Programmed cell death protein 6	E1GGF7	172	76%
C. elegans	HP F59D6.7	213	17561748	*Ascaris suum*	CaBP p22	F1KTJ1	184	69%
C. elegans	bath-25	364	Q9TZM5					
C. elegans	F59D6.7	195	O16343	*Ascaris suum*	CaBP p22	F1KTJ1	184	66%
C. briggsae	HP CBG15199	233	A8XLP7	*Loa loa*	Uncharacterized	E1G817	184	30%
C. remanei	HP CRE_16173	235	E3MSP5	*Loa loa*	Putative uncharacterized protein	E1G817	184	29%
C. briggsae	HP CBG03955	173	A8WVU5	*Loa loa*	Programmed cell death protein 6	E1GGF7	172	77%
C. remanei	HP CRE_29149	186	E3N4M5	*Ascaris suum*	Myosin regulatory light chain	F1KZM0	171	51%
C. brenneri	HP CAEBREN_02194	189	G0N5F2	*Ascaris suum*	CaBP	F1LEH3	192	44%
C. remanei	spectrin	171	A8VT39	*Loa loa*	Spna2 protein	E1G0A3	474	94%
C. remanei	HP CRE_03312	170	E3MML2	*Ascaris suum*	Myosin regulatory light chain 1	F1L8H3	170	88%
C. elegans	spc-1	2427	Q21408	*Ascaris suum*	Spectrin alpha chain	F1KPR0	2422	80%
C. remanei	CRE-SPC-1	2370	E3LCP1	*Ascaris suum*	Spectrin alpha chain	F1KPR0	2422	76%
C. remanei	CRE-ATN-1	921	E3LNI9	*Ascaris suum*	Alpha-actinin sarcomeric	F1KR95	901	79%

C. briggsae	CBR-SPC-1	2427	268578695	*Brugia malayi*	Spectrin alpha chain putative	A8PJH9	2423	79%
C. brenneri	CBN-SPC-1	2427	341874657	*Ascaris suum*	Spectrin alpha chain	F1KPR0	2422	80%
C. briggsae	CBR-MLC-2.2	170	G2J6D1	*Ascaris suum*	Myosin regulatory light chain 1	F1L8H3	170	88%
C. briggsae	CBR-NCS-2	190	A8WWQ4	*Ascaris suum*	NCS-2	F1LFQ0	190	98%
C. briggsae	CBR-NCS-3	174	A8X5Q4	*Ascaris suum*	NCS-1	F1L8J7	208	66%
C. briggsae	Uncharacterized	196	A8X9R8	*Ascaris suum*	CaBP p22	F1KTJ1	184	57%
C. briggsae	CBR-PAT-10	161	A8XBR6	*Onchocerca volvulus*	EF-hand family protein	C7DRP1	161	93%
C. briggsae	CBR-CMD-1	149	A8WPJ8	*Dugesia japonica*	CaM	Q5H765	149	100%
C. briggsae	Uncharacterized	224	A8XQM5	*Loa loa*	Putative uncha-racterized protein	E1GMR4	179	44%
C. briggsae	Uncharacterized	199	A8WYJ2	*Ascaris suum*	CaBP p22	F1KTJ1	184	66%
C. briggsae	Uncharacterized	456	A8X2U3	*Ascaris suum*	EF-hand domain-containing family member A2	F1L0Z4	439	57%
C. briggsae	Uncharacterized	81	A8XEQ0	*Nicotiana tabacum*	CaM NtCaM13	Q94IG4	150	38%
C. brenneri	Troponin C	37	B3GD87	*Anisakis simplex*	Troponin-like protein	Q9U3U5	161	89%
C. elegans	Uncharacterized	176	D1P8J5	*Trypanosoma brucei gambiense*	CaMputative	D0A9H8	148	42%
C. elegans	Uncharacterized	196	Q9N2Y1	*Ascaris suum*	CaBP p22	F1KTJ1	184	55%
C. elegans	Protein F21A10.1a	224	Q9XXN7	*Loa loa*	Putative uncha-racterized protein	E1GMR4	179	43%
C. elegans	Protein Y48B6A.6a	687	Q9U2A6	*Loa loa*	Putative uncha-racterized protein	E1FQF8	210	66%
C. elegans	Uncharacterized	97	Q3V5K4	*Zea mays*	Putative uncha-racterized protein	C4JBJ3	183	39%

C. elegans	Protein Y48B6A.6c	179	A8XMZ5	*Loa loa*	Uncharacterized protein	E1FQF8	210	68%
C. elegans	Protein Y48B6A.6b	203	Q8I0Z7	*Loa loa*	Putative uncha-racterized protein	E1FQF8	210	66%
C. elegans	Uncharacterized	43	Q2L6W4	*Ascaris suum*	CaM	F1LFS6	147	60%
C. elegans	Protein C56A3.6a	477	Q18874	*Ascaris suum*	EF-hand domain-containing family member A2	F1L0Z4	439	52%
C. elegans	NCS family protein-3	199	Q95ZR9	*Ascaris suum*	NCS-1	F1L8J7	208	78%
C. elegans	Protein F21A10.1b	145	A6PVB9	*Loa loa*	Putative uncha-racterized protein	E1GMR4	179	44%
C. elegans	Putative uncharacterized	236	O76670	*Loa loa*	Putative uncha-racterized protein	E1G817	184	31%
C. briggsa	CAEBR CBR-ATN-1	894	A8Y3R8	*Ascaris suum*	Alpha-actinin sarcomeric	F1KR95	901	81%
C. elegans	CAEEL Alpha-Actinin	910	Q9TXC0	*Ascaris suum*	Alpha-actinin sarcomeric	F1KR95	901	79%
C. elegans	CAEEL	894	Q9XVU8	*Ascaris suum*	Alpha-actinin sarcomeric	F1KR95	901	81%
C. elegans	CAEEL	920	Q23158	*Ascaris suum*	Alpha-actinin sarcomeric	F1KR95	901	79%
C. briggsae	CAEBR CBR-PLC-4	752	A8X1E0	*Loa loa*	Phosphatidylinositol-specific PLC	E1FPG2	731	51%
C. elegans	CAEEL PLC	751	Q21754	*Loa loa*	Phosphatidylinositol-specific PLC	E1FPG2	731	51%
C. briggsae	CAEBR Uncharacterized	261	A8Y4A7	*Trichinella spiralis*	Putative thyroglobulin type-1 repeat-containing domain protein	E5S6P2	490	37%
C. briggsae	CAEBR Uncharacterized	143	A8WWN1	*Loa loa*	Calcium and integrin binding	E1G3G4	201	86%

					family member 2			
C. briggsae	CAEBR Uncharacterized	195	A8X8U9	*Ascaris suum*	CaBP p22	F1KTJ1	184	73%
C. briggsae	CAEBR CBR-CBN-1	710	A8X3T5	*Onchocerca volvulus*	CBP-1	Q9NJD9	542	58%
C. elegans	CAEEL CaBP homologue protein 1	585	Q9TZ48	*Onchocerca volvulus*	CBP-1	Q9NJD9	542	59%
C. elegans	CAEEL Protein	260	Q22157	*Trichinella spiralis*	Putative thyroglobulin type-1 repeat-containing domain protein	E5S6P2	490	39%
C. elegans	CAEEL CaBP	482	Q9TZ47	*Onchocerca volvulus*	CBP-1	Q9NJD9	542	60%
C. elegans	CAEEL CaBP	462	Q86S18	*O. volvulus*	CBP-1	Q9NJD9	542	61%
C. elegans	CAEEL Protein	195	Q23643	*Ascaris suum*	CaBP p22	F1KTJ1	184	73%
C. elegans	CAEEL CaBP	679	Q59DM1	*Onchocerca volvulus*	CBP-1	Q9NJD9	542	59%
C. briggsae	CBR-EHS-1	794	A8WU59	*Ascaris suum*	Epidermal growth factor receptor substrate	F1KWZ5	752	40%
C. briggsae	CBR-REPS-1	252	A8WJ38	*Loa loa*	Putative uncha-racterized protein	E1FLG4	651	69%
C. briggsae	CBR-RME-1	611	A8XUA4	*Ascaris suum*	EH domain-containing protein 1	F1L014	552	85%
C. briggsae	CBR-ITSN-1	1099	A8WN16	*Ascaris suum*	Intersectin-1	F1KQL1	1,619	38%
C. elegans	CAEEL RME	786	Q9N5B7	*Ascaris suum*	EH domain-containing protein 1	F1L014	552	85%
C. elegans	CAEEL RME	613	Q8WSP1	*Ascaris suum*	EH domain-containing protein 1	F1L014	552	85%
C. elegans	CAEEL RME	555	Q86S80	*Ascaris suum*	EH domain-containing protein 1	F1L014	552	85%

C. elegans	CAEEL RME	589	Q9N5B9	*Ascaris suum*	EH domain-containing protein 1	F1L014	552	85%
C. elegans	CAEEL RME	835	Q9N5B8	*Ascaris suum*	EH domain-containing protein 1	F1L014	552	85%
C. elegans	CAEEL	1085	Q9U2T9	*Ascaris suum*	Intersectin-1	F1KQL1	1,619	39%
C. elegans	CAEEL RME	576	Q966F0	*Ascaris suum*	EH domain-containing protein 1	F1L014	552	84%
C. elegans	CAEEL EHS-1	796	Q9BIF4	*Ascaris suum*	Epidermal growth factor receptor substrate	F1KWZ5	752	39%
C. elegans	CAEEL	410	Q8MYM6	*Loa loa*	Putative uncharacterized protein	E1FLG4	651	35%
C. briggsae	CBR-SPC-1	2467	A8XJE3	*Ascaris suum*	Spectrin alpha chain	F1KPR0	2422	79%

CaBP- Calcium Binding Protein; CaM – Calmodulin; RME - Receptor Mediated Endocytosis; NCS – Neuronal Calcium Sensor.
