# Supplementary material for: In Silico Prediction and Analysis of Caenorhabditis EF-hand Containing Proteins
Source: PLoS One. 2012 May 25;7(5):e36770. doi: 10.1371/journal.pone.0036770 (PMC3360750; doi:10.1371/journal.pone.0036770)
Supplement: Table S4 — Calcium binding constant of EF-hand CaBP. (PDF) [file pone.0036770.s004.pdf]

Table S4. Calcium binding constant of EF-hand CaBP

UniprotID/gi	Predicted Name	EF-hands	EF-hand Sequence	Ca^{+2} binding affinity
Q18092_1	C18E9.1	4	DKDGNGSISSKE	strong
Q18092_2			DIDGSGTIDFGE	strong
Q18092_3			DRDGNGFITADE	strong
Q18092_4			DIDGDGQIDYEE	strong
G0NP24_1	HP CAEBREN_11606	4	DKDGNGSISSKE	strong
G0NP24_2			DIDGSGTIDFGE	strong
G0NP24_3			DRDGNGFITADE	strong
G0NP24_4			DIDGDGQIDYEE	strong
E3LG34_1	CBR-CAL-2	4	DKDGNGSISSKE	strong
E3LG34_2			DIDGSGTIDFGE	strong
E3LG34_3			DRDGNGFITADE	strong
E3LG34_4			DIDGDGQIDYEE	strong
E3LNK3_1	CRE-CAL-1	4	DKDGNGTISTKE	strong
E3LNK3_2			DIDGNGQIEFPE	strong
E3LNK3_3			DKDGNGVITAQE	strong
E3LNK3_4			DVDGDGEIDYEE	strong
P04630_1	CAL-1	4	DKDGNGTISTKE	strong
P04630_2			DIDGNGQIEFPE	strong
P04630_3			DKDGNGVITAQE	strong
P04630_4			DVDGDGEIDYEE	strong
G0MP03_1	HP CAEBREN_30810	4	DKDGNGTISTKE	strong
G0MP03_2			DIDGNGQIEFPE	strong
G0MP03_3			DKDGNGVITAQE	strong
G0MP03_4			DVDGDGEIDYEE	strong
268557584_1	CBR-CAL-1	4	DKDGNGTISTKE	strong
268557584_2			DIDGNGQIEFPE	strong
268557584_3			DKDGNGVITAQE	strong
268557584_4			DVDGDGEIDYEE	strong
268552973_1	CBR-CAL-3	4	DKDGNGTISIKE	strong
268552973_2			DLDGNGQVEFPE	strong
268552973_3			DRDGNGVITANE	strong
268552973_4			DCDGNGEIDYEE	strong
E3M5U3_1	CRE-CAL-3 protein	4	DKDGNGTISIKE	strong
E3M5U3_2			DLDGNGQVEFPE	strong
E3M5U3_3			DRDGNGVITANE	strong
E3M5U3_4			DCDGNGEIDYEE	strong
G0P5R9_1	HP CAEBREN_12026	4	DKDGNGTISIKE	strong
G0P5R9_2			DLDGNGQVEFPE	strong
G0P5R9_3			DRDGNGVITANE	strong
G0P5R9_4			DCDGNGEIDYEE	strong
A8XBN2_1	CBR-CAL-3	4	DKDGNGTISIKE	strong

A8XBN2_2			DLDGNGQVEFPE	strong
A8XBN2_3			DRDGNGVITANE	strong
A8XBN2_4			DCDGNGEIDYEE	strong
E3M5L0_1	CBR-CAL-4	4	DKDGNNTMNIKE	strong
E3M5L0_2			DVDGNGKIDFGE	Strong
E3M5L0_3			DKDGNGYITAQ	Strong
E3NKA0_1	HP CRE_15028	3	DKDGNNTMNIKE	strong
E3NKA0_2			DVDGNGKIDFGE	strong
E3NKA0_3			DKDGNGYITAQE	strong
O18058_1	CAL-4	3	DKDGNNTMNIKE	strong
O18058_2			DVDGNGKIDFGE	strong
O18058_3			DKDGNGYITAQE	strong
C6KRM4_1	T07G12.1b	3	DKDGNNTMNIKE	strong
C6KRM4_2			DVDGNGKIDFGE	strong
C6KRM4_3			DKDGNGYITAQE	strong
C6KRM3_1	T07G12.1a	3	DKDGNNTMNIKE	strong
C6KRM3_2			DVDGNGKIDFGE	strong
C6KRM3_3			DKDGNGYITAQE	strong
Q94288_1	CaM related genes protein 3	4	DKDGNGTISIKE	strong
Q94288_2			DLDGNGQVEFPE	strong
Q94288_3			DRDGNGVITANE	strong
Q94288_4			DCDGNGEIDYEE	strong
115534350_1	CAL-3	4	DKDGNGTISIKE	strong
115534350_2			DLDGNGQVEFPE	strong
115534350_3			DRDGNGVITANE	strong
115534350_4			DCDGNGEIDYEE	strong
341889270_1	HP CAEBREN_06721	2	DADRSGAIAIDE	strong
341889270_2			DVDGNGVIDYDE	strong
E3LNL5_1	HP CRE_27132	4	DADRSGAIAIDE	strong
E3LNL5_2			DVDGNGVIDYDE	strong
P90802_1	HP E02A10.3	4	DADRSGAIAIDE	strong
P90802_2			DVDGNGVIDYDE	strong
A8Y3P8_1	HP CBG23534	4	DADRSGAIAIDE	strong
A8Y3P8_2			DVDGNGVIDYDE	strong
Q09665_1	tnc-2	4	DADGSGEIEFEE	strong
Q09665_2			DADGSGTVDFDE	strong
A8WSS9_1	CRE-TNC-2	4	DADGSGEIEFEE	strong
A8WSS9_2			DADGSGTVDFDE	strong
Q11083_1	HP B0563.7	4	DTDGSGAIGNEE	strong
Q11083_2			DADGNGEIDFEE	strong
Q11083_3			DQDRNGIITENE	strong
Q95XF6_1	HP Y73B3A.12	4	DADGNGTIDFPE	strong
Q95XF6_2			DKDGNGFISAAE	strong

E3MRT8_1	HP CRE_14767	4	DTDGSGAIGNEE	strong
E3MRT8_2			DADGNGEIDFEE	strong
E3MRT8_3			DQDRNGIITENE	strong
268580583_1	HP CBG00175	4	DTDGSGAIGNEE	strong
268580583_2			DADGNGEIDFEE	strong
268580583_3			DQDRNGIITENE	strong
268531200_1	HP CBG02410	4	DKNGDGRITRQE	strong
268531200_2			DLDGNGCIDIDE	strong
268531200_3			DKNGDGMISIDD	Strong
268531200_4			DLDHDGMIDFQE	strong
Q09980_1	HP F12A10.5	4	DKNGDGRITRQE	strong
Q09980_2			DLDGNGCIDIDE	strong
Q09980_3			DKNGDGVISIDD	strong
Q09980_4			DLDHDGMIDFQE	strong
E3M4N3_1	HP CRE_11951	4	DKNGDGRITRQE	strong
E3M4N3_2			DLDGNGCIDIDE	strong
E3M4N3_3			DKNGDGVISIDD	strong
E3M4N3_4			DLDHDGMIDFQE	strong
A8WME8_1	HP CBG_00175	4	DTDGSGAIGNEE	strong
A8WME8_2			DADGNGEIDFEE	strong
A8WME8_3			DQDRNGIITENE	strong
A8WUD8_1	HP CBG_02410	4	DKNGDGRITRQE	strong
A8WUD8_2			DLDGNGCIDIDE	strong
A8WUD8_3			DKNGDGMISIDD	strong
B3GEK4_1	troponin C	4	DADGSGKLEFDE	medium
B3GEK4_2			DEDGSGKIEFEE	strong
E3MX54_1	CRE-PAT-10	4	DADGSGKLEFDE	medium
E3MX54_2			DEDGSGKIEFEE	strong
A8XC16_1	HP CBG_11013	4	DANNDGRITIDE	strong
A8XC16_2			DNDQNGVITFDE	strong
A8XC16_3			DKDKDGFIEKME	strong
A8XC16_4			DIDGDGKISFEE	strong
Q18136_1	Vitellogenin-linked	4	DLNGDGYIQREE	strong
Q18136_2			DKDCDGNIDFQE	strong
Q18136_3			DVDGDGYITRSE	strong
Q18136_4			DQNNDGKINFQE	strong
G0M8G3_1	CBN-CAL-5	4	DLNGDGFIQREE	strong
G0M8G3_2			DKDCDGNIDFQE	strong
G0M8G3_3			DVDGDGYITRSE	strong
G0M8G3_4			DQNNDGKINFQE	strong
G0N500_1	HP CAEBREN_26130	4	DANNDGRITIDE	strong
G0N500_2			DNDQNGVITFDE	strong
G0N500_3			DKDKDGFIEKME	strong
G0N500_4			DIDGDGKISFEE	strong

E3LE72_1	CRE-CAL-5	4	DLNGDGFIQREE	strong
E3LE72_2			DKDHDGNIDFQE	strong
E3LE72_3			DVDGDGYITRSE	strong
E3LE72_4			DQNNDGKINFQE	strong
Q21165_1	K03A1.4 a	4	DANNDGRITIDE	strong
Q21165_2			DNDQNGVITFDE	strong
Q21165_3			DKDKDGFIEKME	strong
Q21165_4			DIDGDGKISFEE	strong
A8XK99_1	CBR-UVT-2	4	DLNGDGFIQREE	strong
A8XK99_2			DKDHDGNIDFQE	strong
A8XK99_3			DVDGDGYITRSE	strong
A8XK99_4			DQNNDGKINFSE	strong
E3LD20_1	HP CRE_00078	4	DANNDGRITIDE	strong
A8XK99_2			DNDQNGVITFDE	strong
A8XK99_3			DKDKDGFIEKME	strong
A8XK99_4			DIDGDGKISFEE	strong
268580069_1	HP CBG11013	4	DANNDGRITIDE	strong
268580069_2			DNDQNGVITFDE	strong
Q09510	mlc-4	3	DQNRDGFIDQED	strong
268580069_3<br>268580069_4<br>115533480_1	 <br>HP K03A1.4	 <br>4	DKDKDGFIEKME<br>DIDGDGKISFED<br>DANNDGRITIDE	strong<br>strong<br>strong
115533480_2			DNDQNGVITFDE	strong
115533480_3			DKDKDGFIEKME	strong
G0MQV0_1	HP CAEBREN_16765	3	DADGNGEIDFEE	strong
G0MQV0_2			DQDRNGIITENE	strong
A8X9U2	CBR-MLC-4	1	DQNRDGFIDQED	strong
G0MKR0	HP CAEBREN_07889	2	DQNRDGFIDQDD	strong
G0PGC7_1	HP CAEBREN_10686	4	DVDGSGSLSVEE	medium
G0PGC7_2			DEDGNGEVDFRE	strong
G0PGC7_3			DMDRDGFISNGE	strong
G0PGC7_4			DKDGDGKISFQE	strong
Q20804_1	CNB-1	4	DVDGSGSLSVEE	medium
Q20804_2			DEDGNGEVDFRE	strong
Q20804_3			DMDRDGFISNGE	strong
Q20804_4			DKDGDGKISFQE	strong
E3MQD1_1	CRE-CNB-1	4	DVDGSGSLSVEE	medium
E3MQD1_2			DEDGNGEVDFRE	strong
E3MQD1_3			DMDRDGHISNGE	strong
E3MQD1_4			DKDGDGKISFQE	strong
E3LTC7	CRE-MLC-4	3	DQNRDGFIDQED	strong
A8XK57_1	HP CBG14543	4	DVDKDGRLSRNE	strong
A8XK57_2			DTDKTGKISKEE	strong
A8XK57_3			DSDGDGAITEDE	strong

A8XK57_4			DLNGDGKITFFE	strong
A8XVF9	HP CBG_19361	4	DEDCDGLIETEE	strong
Q20358_1	HP F43C9.2	4	DVDKDGRLSRNE	strong
Q20358_2			DTDKTGKISKEE	strong
Q20358_3			DSDGDGAITEDE	strong
Q20358_4			DLNGDGKITFFE	strong
341874474_1	HP CAEBREN_15606	4	DVDKDGRLSRNE	strong
341874474_2			DTDKTGKISKEE	strong
341874474_3			DSDGDGAITEDE	strong
341874474_4			DLNGDGKITFFE	strong
E3LEB5_1	HP CRE_00789	4	DVDKDGRLSRNE	strong
E3LEB5_2			DTDKTGKISKEE	strong
E3LEB5_3			DSDGDGAITEDE	strong
E3LEB5_4			DLNGDGKITFFE	strong
268559254	HP CBG19361	1	DEDCDGLIETEE	strong
G0NPN1	HP CAEBREN_20342	1	DEDCDGLIETDD	strong
E3MHC8	HP CRE_22883	2	DEDCDGLIETDD	strong
17560804	HP F40F9.8	5	DEDSDGLIETDD	strong
E3N7H9_1	HP CRE_13655	4	DKDRSGQISSDE	strong
E3N7H9_2			DSNGDGAINFSE	strong
E3N7H9_3			DTDGSGNIDKTE	strong
E3N7H9_4			DRSHSNRINFDD	medium
Q95Y96_1	HP M04F3.4	5	DKDRSGQISSDE	strong
Q95Y96_2			DSNGDGAINFQE	strong
Q95Y96_3			DTDGSGNIDKSE	strong
Q95Y96_4			DRSHSNRINFDD	medium
351050110_1	M04F3.4 a	4	DKDRSGQISSDE	strong
351050110_2			DSNGDGAINFQE	strong
351050110_3			DTDGSGNIDKSE	strong
351050110_4			DRSHSNRINFDD	medium
17561748_1	HP F59D6.7	2	DLNKNNYITREE	strong
17561748_2			DQDRDGKISFED	strong
Q9TZM5_1	bath-25	4	DKDGDGFISVAE	strong
Q9TZM5_2			DISGDGLVDLEE	medium
O16343_1	F59D6.7	5	DLNKNNYITREE	strong
O16343_2			DQDRDGKISFED	strong
A8XLP7_1	HP CBG15199	6	DADNDGKLSEKE	strong
A8XLP7_2			DLDRNGHLDISE	strong
A8XLP7_3			DSNGDNRLDQLE	strong
A8XLP7_4			DKNQDGYLTENE	strong
E3MSP5_1	HP CRE_16173	5	DANNDGKLSEKE	strong
E3MSP5_2			DLDRNGHLDISE	strong
E3MSP5_3			DLNDDGIVDKAE	medium
E3MSP5_4			DVNQDGQLTYEE	strong

E3MSP5_5			DSNGDNRLDQLE	medium
E3MSP5_6			DKNQDGYLQENE	medium
A8WVU5_1	HP CBG03955	4	DKDRSGQISADE	strong
A8WVU5_2			DSNGDGAINFQE	strong
A8WVU5_3			DTDGSGNIDKTE	strong
A8WVU5_4			DRSHSNRINFDD	medium
E3N4M5	HP CRE_29149	4	DQNRDGLIDQDD	medium
G0N5F2_1	HP CAEBREN_02194	3	DDDGSGTISSSE	strong
G0N5F2_2			DVSGDGQIDFEE	strong
G0N5F2_3			DKNPEKVISAEE	medium
A8VT39	Spectrin	2	DPNRDGYVTLQE	strong
E3MML2	HP CRE_03312	2	DQNKDGVIDKSD	medium
Q21408	SPC-1	2	DPNRDGYVTLQE	medium
E3LCP1	CRE-SPC-1	2	DPNRDGYVTLQE	medium
268578695	CBR-SPC-1	2	DPNRDGYVTLQE	medium
341874657	CBN-SPC-1	2	DPNRDGYVTLQE	medium
G2J6D1	CBR-MLC-2.2	3	DQNKDGVIDKSD	medium
A8WWQ4_1	CBR-NCS-2	4	DTDNSGFIDFKE	strong
A8WWQ4_2			DIDGNGTIDEKE	strong
A8WWQ4_3			DVNNDKELTLKE	medium
A8X5Q4	CBR-NCS-3	2	DVDKDGFITKDE	medium
A8X9R8_1	Uncharacterized	4	DLNKSGTITKDE	Medium
A8X9R8_2			DRDGDGYITFQE	strong
A8XBR6_1	CBR-PAT-10	4	DADGSGKLEFDE	medium
A8XBR6_2			DEDGSGKIEFEE	strong
A8WPJ8_1	CBR-CMD-1	3	DKDGDGTITTKE	strong
A8WPJ8_2			DADGNGTIDFPE	strong
A8WPJ8_3			DKDGNGFISAAE	strong
A8WPJ8_4			DIDGDGQVNYEE	strong
A8XQM5	Uncharacterized	3	DNDGNGTINFQE	strong
A8WYJ2_1<br>A8WYJ2_2	Uncharacterized	3	DEDDNGNIDRDE<br>DENNDGTLSYPE	strong<br>medium
A8X2U3	Uncharacterized	4	DEDDNGNIDRDE	strong
A8XEQ0_1	Uncharacterized	4	DADGSGKLEFDE	medium
A8XEQ0_2			DEDGSGKIEFEE	strong
B3GD87	Troponin C	2	DEDGSGKIEFEE	strong
D1P8J5_1	Uncharacterized	2	DEDGNGFISVAE	strong
D1P8J5_2			DIDRNGQVDLDE	strong
Q9N2Y1_1	Uncharacterized	3	DLNKSGTITKDE	medium
Q9N2Y1_2			DRDGDGFITFQE	strong
Q9XXN7	F21A10.1a	2	DNDGNGTINFQE	strong
Q9U2A6	Y48B6A.6a	2	DEDQDGKISQRE	strong
Q3V5K4_1	Uncharacterized	4	DEDGNGFISVAE	strong
Q3V5K4_2			DIDRNGQVDLDE	strong

A8XMZ5	Y48B6A.6c	2	DEDQDGKISQRE	strong
Q8I0Z7	Y48B6A.6b	1	DEDQDGKISQRE	strong
Q18874_1	C56A3.6a	4	DEDDNGNIDRDE	strong
Q18874_2			DENNDGTLSYPE	medium
Q95ZR9_1	calcium sensor 3	2	DDNHDGHISFSE	strong
Q95ZR9_2			DVDKDGFITKDE	strong
A6PVB9	F21A10.1b	3	DNDGNGTINFQE	strong
O76670_1	Uncharacterized	6	DANNDGKLSEKE	medium
O76670_2			DLDRNGHLDISE	strong
O76670_3			DLNDDGIVDKA	medium
O76670_4			DTNRDGQLTYAE	medium
O76670_5			DANGDNKLDQLE	medium
O76670_6			DKNQDGYLTENE	medium
A8X1E0	CBR-PLC-4	1	DTNKNGLLSFDE	medium
Q21754	PLC4	3	DTNKNGLLSFDE	medium
A8Y4A7	Uncharacterized	3	DMDRDRKLTFEE	strong
A8X8U9_1	Uncharacterized	5	DLNKNDYITREE	strong
A8X8U9_2			DADRDGKISFEE	strong
A8X3T5_1	CBR-CBN-1	8	DTNHDNVLDARE	medium
A8X3T5_2			DTDRDGLLSLQE	strong
A8X3T5_3			DLNGDGHISVDE	medium
A8X3T5_4			DADNSGTLTLDE	medium
A8X3T5_5			DTNGDGLVSTAE	medium
A8X3T5_6			DLDGNDAVTFDE	strong
Q22157	T04F3.2	8	DLNKDRKLTFDE	medium
Q23643_1	ZK856.8	2	DLNKNDYITREE	Strong
Q23643_2			DADRDGKISFDE	strong
A8WU59	CBR-EHS-1	1	DQDKDGSLDRIE	strong
A8WJ38	CBR-REPS-1	2	DVNEDGWLDLQE	medium
A8XUA4	CBR-RME-1	1	DIDKDGQLDADE	strong
A8WN16	CBR-ITSN-1	1	DLDKDGRLDIRE	strong
Q9N5B7	RME-1, isoform a	1	DIDKDGQLDADE	strong
Q8WSP1	RME-1, isoform e	1	DIDKDGQLDADE	strong
Q86S80	RME-1, isoform f	1	DIDKDGQLDADE	strong
Q966F0	RME-1, isoform d	1	IDKDGQLDADE	Medium
Q9N5B8	RME-1, isoform b	2	DIDKDGQLDADE	strong
Q9U2T9_1	Y116A8C.36a	2	DLDKDGRLDIRE	strong
Q9U2T9_2			DVNKDGKLSVDE	medium
Q9BIF4	EHS-1	1	DQDKDGNLDRIE	strong
Q8MYM6	Y39B6A.38	1	DVNEDGWLDLNE	medium

CaBP- Calcium Binding Protein; HP - Hypothetical Protein; PLC- Phospholipase C; RME – Receptor Mediated Endocytosis; PAT 10 - Paralysed arrest at two-fold protein 10; NCS - Neuronal Calcium Sensor, MLC- Myosin Light chain; SPC- Spectrin; EF hand no.– Number of EF hands.
